# Identification of imidazo[4,5-c]pyridin-2-one derivatives as novel Src family kinase inhibitors against glioblastoma

**DOI:** 10.1080/14756366.2021.1948542

**Published:** 2021-07-08

**Authors:** Lishun Zhang, Zichao Yang, Huiting Sang, Ying Jiang, Mingfeng Zhou, Chuan Huang, Chunhui Huang, Xiaoyun Wu, Tingting Zhang, Xingmei Zhang, Shanhe Wan, Jiajie Zhang

**Affiliations:** a Guangdong Provincial Key Laboratory of New Drug Screening, School of Pharmaceutical Sciences, Southern Medical University, Guangzhou, PR China; b Guangdong Provincial Key Laboratory of New Drug Design and Evaluation, Guangzhou, PR China; c Department of Neurobiology, Guangdong Province Key Laboratory of Psychiatric Disorders, School of Basic Medical Sciences, Southern Medical University, Guangzhou, PR China

**Keywords:** Glioblastoma, Src family kinase, imidazo[4,5-c]pyridin-2-one, kinase inhibitor, molecular simulation

## Abstract

Glioblastoma multiforme (GBM) is the most common and malignant primary brain tumour in the central nervous system (CNS). As the ideal targets for GBM treatment, Src family kinases (SFKs) have attracted much attention. Herein, a new series of imidazo[4,5-c]pyridin-2-one derivatives were designed and synthesised as SFK inhibitors. Compounds **1d**, **1e**, **1q**, **1s** exhibited potential Src and Fyn kinase inhibition in the submicromolar range, of which were next tested for their antiproliferative potency on four GBM cell lines. Compound **1s** showed effective activity against U87, U251, T98G, and U87-EGFRvIII GBM cell lines, comparable to that of lead compound PP2. Molecular dynamics (MDs) simulation revealed the possible binding patterns of the most active compound **1s** in ATP binding site of SFKs. ADME prediction suggested that **1s** accord with the criteria of CNS drugs. These results led us to identify a novel SFK inhibitor as candidate for GBM treatment.

## Graphical Abstract

**Figure:**
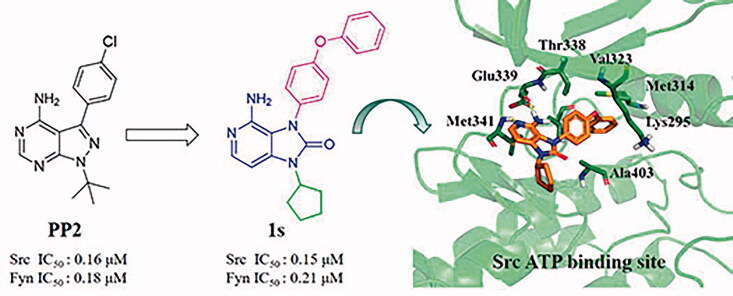

## Introduction

Glioblastoma multiforme (GBM) is the most common and aggressive brain tumours in adults^1^. Even with high-intensity treatments, the prognosis of GBM patients remains poor, with median survival less than 15 months^2^^,^^3^. Multiple signalling pathways have become potential therapeutic targets due to their abnormal expression in GBM. In particular, deregulated SFKs signalling plays a key role in GBM development^4–6^.

Src family kinases (SFKs) are a group of non-receptor tyrosine kinases composed of 11 members, including eight main members, such as c-Src (Src), Fyn, Yes, Fgr, Lyn, Blk, Hck, and Lck^7^^,^^8^. They share a set of conserved domains which includes an N-terminal Src homology domain (SH4), followed by a unique domain, two Src homology domains (SH3 and SH2), a catalytic kinase domain (SH1), and a C-terminal regulatory tail^9^. SFKs are highly expressed in GBM cell lines and tumour samples, in which abnormal activation of SFKs induced multiple tumour-promoting effects, including reducing cell apoptosis, increasing angiogenesis, promoting cell proliferation, motility as well as invasion^10–12^. In detail, Yes and phosphatidylinositol 3-kinase bind prototypical death receptor CD95 to mediate invasion of GBM^13^. Hck stimulates GBM progression *via* mediating epithelial-mesenchymal transition process^14^. Lyn enhances GBM cell survival by promoting autophagy under nutrient deprivation^15^. More importantly, both Src and Fyn are downstream targets of EGFR oncogenic signalling, and their overexpression is frequently occurred in GBM patients. Glioblastoma exhibiting activated EGFR signalling also showed the activation of Src and Fyn, which indicates that Src and Fyn inhibition may improve the efficacy of anti-EGFR targeted therapy^16–18^.

**Figure 1.**
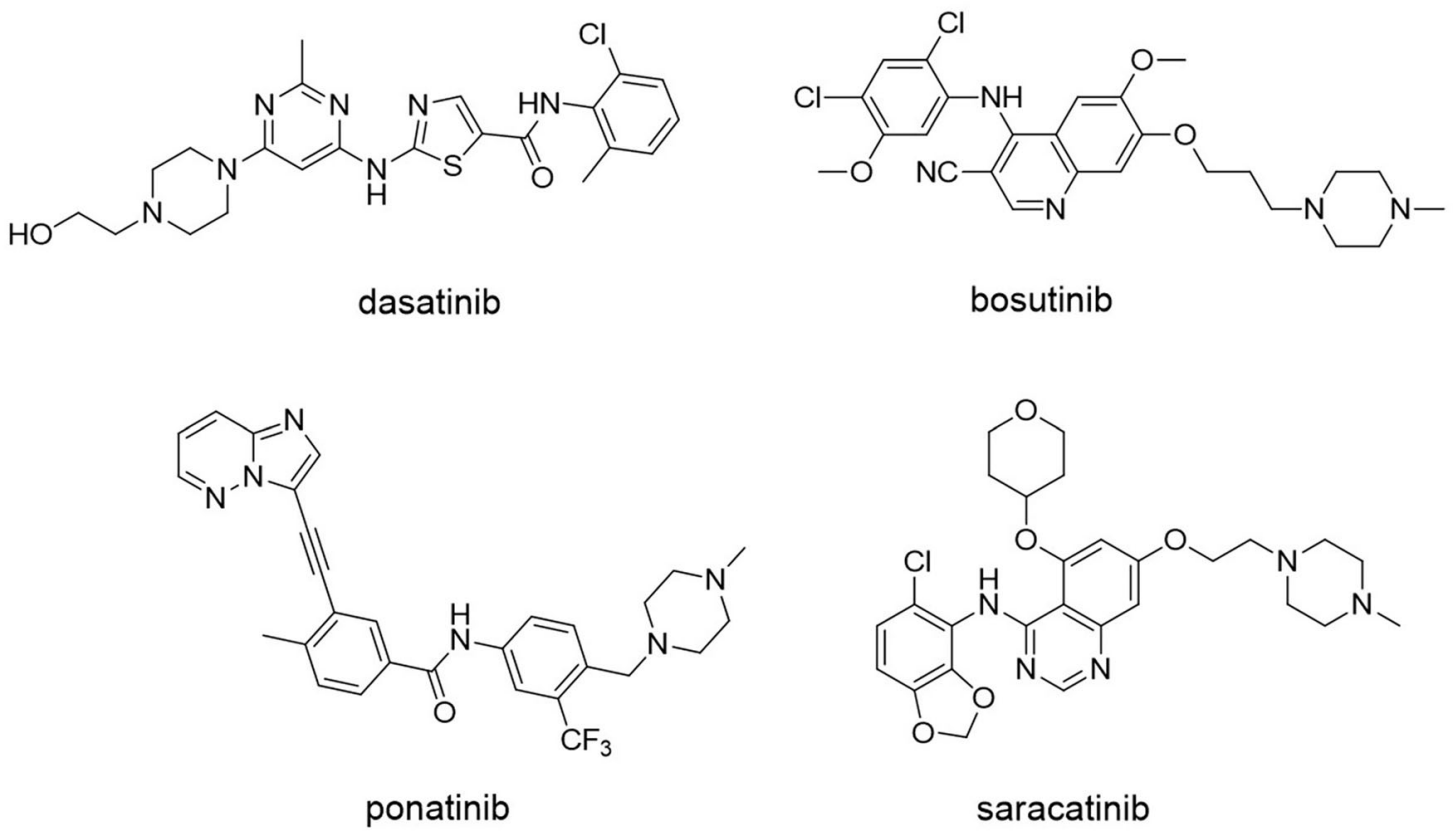
Chemical structures of approved SFK inhibitors.

For all these reasons, SFKs can be used as the potential targets in GBM therapy. Up to now, almost all reported SFK inhibitors are ATP competitive inhibitors. Due to the high conservation of ATP binding sites, it is inevitable that these inhibitors not only inhibit SFKs but also other kinases, presenting as non-strict SFK inhibitors^19^. However, this non-strict selectivity may be beneficial for tumour therapy, which depends on the pathological features of tumours^20^. Four small molecule SFK inhibitors have been approved by FDA, i.e. dasatinib, ponatinib, bosutinib, and saracatinib (Figure 1)^21^. They were initially developed as Src/Abl or pan-kinase inhibitors, and currently dasatinib (Sprycel^®^, Bristol-Myers Squibb, New York, NY approved in 2006) , ponatinib (Iclusig^®^, Ariad Pharmaceuticals, Cambridge, MA approved in 2012), bosutinib (Bosulif^®^, Wyeth, Madison, NJ approved in 2012) are currently mainly used for the treatment of haematological malignancies^22–25^. Saracatinib (AstraZeneca, Cambridge, UK approved in 2019) was recently awarded the orphan drug qualification for the treatment of idiopathic pulmonary fibrosis (IPF) by FDA and is undergoing clinical trials for several solid tumours^26^.

Failure of targeted therapies and resistance to chemoradiotherapy is often attributed to the broad cellular heterogeneity of GBM. This large heterogeneity often leads to compensatory activation of associated cellular signalling pathways to evade treatment. Studies from *in vitro* and preclinical studies have confirmed the importance of Src inhibitor in combination with radiotherapy and proton therapy for GBM, reporting that Src inhibition enhances the radiotherapy and proton therapy efficacy against GBM^27–29^. These encouraging results inspire us that the development of SFK inhibitors may also provide an important means for tumour radiosensitisation.

Although a large number of preclinical studies have shown good prospects for small molecule SFK inhibitors in the treatment of GBM, the clinical trial effect is not optimistic^30^^,^^31^. The reasons for the failure of these clinical studies are mainly attributed to the obstruction of blood–brain barrier (BBB) in the central nervous system (CNS)^32^^,^^33^. Therefore, SFK inhibitors conforming to the drug characteristics of the CNS are also crucial for the treatment of GBM.

Kinase inhibitors usually contain an aromatic heterocyclic core that can form H bond with the ATP binding site, which is essential to ensure good kinase activity^34^. For example, PP2, a well-known small molecule SFK inhibitor, contains a pyrazolo[3,4-d]pyrimidine scaffold, which was first reported in 1996 and widely used as lead compound for research^35^. In this work, a novel imidazo[4,5-c]pyridin-2-one core was synthesised as the starting point for the development of new ATP pocket binder. At the kinase level, we evaluated the inhibitory activity of the synthesised compounds against the Src and Fyn, two most important members in GBM pathogenesis. Then, the compounds with the best enzyme inhibition activity were cultured with U87, U251, T98G, and U87-EGFRvIII GBM cell lines to evaluate their antiproliferative activity. Molecular dynamics simulation was carried out to analyse the binding mode of imidazo[4,5-c]pyridin-2-one derivatives with SFKs. ADME prediction was employed to assess whether the most active compound accords with the criteria for CNS drugs.

## Materials and methods

### Chemistry

Starting materials and solvents were purchased from commercial supplier without further purification. All oxygen-sensitive or water-sensitive reactions were carried out under nitrogen atmosphere. All reactions were monitored by TLC on 0.25 mm silica gel plate (HSGF254) and observed under 254 nm UV lamp. Compounds were subjected to column chromatography using commercial silica gel (200–300 mesh, Merck, Darmstadt, Germany) through medium-low pressure preparative chromatograph (Yamazen Co., Osaka, Japan). ^1^H NMR and ^13 ^C NMR spectra were recorded on Bruker AVIII 400 MHZ using deuterated dimethyl sulfoxide (DMSO-d_6_) or deuterated chloroform (CDCl_3_). The chemical shift unit was ppm and the internal standard was tetramethylsilane (TMS). The precise molecular weight was determined by Waters ZQ4000 mass spectrometer (Waters Co., Milford, MA) and obtained by positive and negative ion mode scanning using electrospray ionisation (ESI) source.

#### Representative procedure A for the synthesis of compounds 1a–j

##### 2-chloro-N-cyclopentyl-3-nitropyridin-4-amine (3d)

To a solution of **2** (8 g, 41.5 mmol) and Et_3_N (5.4 g, 53.9 mmol) in DMF (60 ml) was added cyclopentylamine (3.5 g, 41.5 mmol). The solution was stirred overnight at room temperature. The resulting solution was diluted with H_2_O (120 ml), extracted with ethyl acetate (120 ml × 3) and the organic layer was combined. The resulting mixture was washed with saturated sodium and dried over by anhydrous sodium sulphate and concentrated. The residue was purified by flash chromatography eluting with 10–50% ethyl acetate in petroleum ether to provide the desired product **3d** as yellow oil (8.5 g, yield: 85%). ^1^H NMR (400 MHz, DMSO-d_6_) δ 8.01 (d, *J* = 6.1 Hz, 1H), 7.14 (d, *J* = 6.7 Hz, 1H), 6.96 (d, *J* = 6.1 Hz, 1H), 3.94 (h, *J* = 6.6 Hz, 1H), 1.96 (td, *J* = 9.4, 5.3 Hz, 2H), 1.66 (q, *J* = 4.3 Hz, 2H), 1.54 (q, *J* = 7.5, 6.6 Hz, 4H). ESI-MS: *m/z* 242.68 [M + H]^+^.

##### N^4^-cyclopentyl-N^2^,N^2^-bis(4-methoxybenzyl)-3-nitropyridine-2,4-diamine (4d)

To a solution of **3d** (8 g, 33.2 mmol) and Et_3_N (4.36 g, 43.2 mmol) in isopropanol (60 ml) was added bis(4-methoxybenzyl)-amine (8.53 g,17.3 mmol), The resulting solution was stirred overnight at 95 °C. The reaction mixture was cooled and concentrated under vacuum. The residue was purified by flash chromatography eluting with 10–50% ethyl acetate in petroleum ether to provide the desired product **4d** as yellow oil (13.8 g, yield: 90%). ^1^H NMR (400 MHz, DMSO-d_6_) δ 7.86 (d, *J* = 5.8 Hz, 1H), 7.48 (d, *J* = 6.7 Hz, 1H), 7.06 (d, *J* = 8.6 Hz, 4H), 6.85 (d, *J* = 8.6 Hz, 4H), 6.33 (d, *J* = 5.9 Hz, 1H), 4.35 (s, 4H), 3.99 − 3.89 (m, 1H), 3.73 (s, 6H), 2.07 − 1.93 (m, 2H), 1.74 − 1.44 (m, 6H). ESI-MS: *m/z* 463.88 [M + H]^+^.

##### N^4^-cyclopentyl-N^2^,N^2^-bis(4-methoxybenzyl)pyridine-2,3,4-triamine (5d)

To a solution of **4d** (13 g, 28.1 mmol) and AcOH (25.3 g, 421.2 mmol) in EtOH/H_2_O (3:1, 100 ml) was added Fe powder (6.29 g, 112.4 mmol), The resulting solution was stirred 2 h at 80 °C and then concentrated under vacuum. The pH value of the solution was adjusted to 9 with sodium bicarbonate. The resulting solution was extracted with ethyl acetate (100 ml × 3) and the organic layers were washed with sodium bicarbonate, filtered and dried over anhydrous sodium sulphate, then concentrated under vacuum. The crude mixture was purified by flash chromatography eluting with 10–50% ethyl acetate in petroleum ether to provide the desired product **5d** as brown solid (11.2 g, yield: 92%). ^1^H NMR (400 MHz, DMSO-d_6_) δ 7.42 (d, *J* = 5.4 Hz, 1H), 7.18 (d, *J* = 8.5 Hz, 4H), 6.81 (d, *J* = 8.5 Hz, 4H), 6.24 (d, *J* = 5.5 Hz, 1H), 5.18 (s, 1H), 4.44 (s, 2H), 1.94 (d, *J* = 15.7 Hz, 2H), 1.74 − 1.63 (m, 2H), 1.60 − 1.45 (m, 4H). ESI-MS: *m/z* 433.85 [M + H]^+^.

##### 4-(bis(4-methoxybenzyl)amino)-1-cyclopentyl-1,3-dihydro-2H-imidazo[4,5-c]pyridin-2-one (6d)

To a solution of **5d** (10 g, 23.1 mmol) in anhydrous THF (60 ml) was added CDI (9.35 g, 57.8 mmol), The resulting solution was stirred overnight at 75 °C. The reaction mixture was cooled and concentrated. The residue was purified by flash chromatography eluting with 10–50% ethyl acetate in petroleum ether to provide the desired product **6d** as brown solid (9.43 g, yield: 89%). ^1^H NMR (400 MHz, DMSO-d_6_) δ 10.85 (s, 1H), 7.79 (d, *J* = 5.4 Hz, 1H), 7.09 (d, *J* = 8.4 Hz, 4H), 6.83 (d, *J* = 8.5 Hz, 4H), 6.78 (d, *J* = 5.5 Hz, 1H), 4.68 (p, *J* = 8.7 Hz, 1H), 4.47 (s, 4H), 3.71 (s, 6H), 2.01 (d, *J* = 5.8 Hz, 2H), 1.91 (d, *J* = 10.2 Hz, 4H), 1.71 − 1.58 (m, 2H). ESI-MS: *m/z* 459.83 [M + H]^+^.

##### 4-amino-3-(4-chlorophenyl)-1-cyclopentyl-1,3-dihydro-2H-imidazo[4,5-c]pyridin-2-one (1d)

To a solution of **6d** (1 g, 2.2 mmol) in DCM (10 ml) was added TFA (2.5 g, 22 mmol), The resulting solution was stirred 4 h at room temperature, The pH value of the solution was adjusted to 9 with sodium bicarbonate. The resulting solution was extracted with dichloromethane (60 ml × 3) and the organic layers combined and dried over anhydrous sodium sulphate. The resulting mixture was concentrated under vacuum to give **7d** as white solid and used without further purification in next step. Then to the flask containing **7d** was added 4-chlorophenylboronic acid (680 mg, 4.4 mmol), Cu(OAc)_2_ (397 mg, 2.2 mmol), pyridine (344 mg, 4.4 mmol), and 4 A molecular sieves (500 mg). The mixture was suspended in DCM and stirred at room temperature under ambient-pressure oxygen atmosphere. After 24 h, the mixture was filtered using ethyl acetate and the solvent was removed under vacuum, then purified by flash chromatography eluting with 20–70% ethyl acetate in petroleum ether to provide the desired product **1d** as brown solid (80 mg, 11% yield for two steps). ^1^H NMR (400 MHz, DMSO-d_6_) δ 7.77 (d, *J* = 5.5 Hz, 1H), 7.60 (d, *J* = 8.1 Hz, 2H), 7.45 (d, *J* = 8.2 Hz, 2H), 6.75 (d, *J* = 5.5 Hz, 1H), 4.82 (s, 2H), 4.77 − 4.65 (m, 1H), 2.14 − 1.76 (m, 6H), 1.75 − 1.54 (m, 2H). ^13 ^C NMR (101 MHz, DMSO-d_6_) δ 152.52, 144.04, 141.51, 135.55, 133.57, 133.02, 130.05, 129.31, 109.73, 97.26, 53.86, 28.97, 24.94. ESI-MS: *m/z* 329.73 [M + H]^+^.

##### 4-amino-3-(4-chlorophenyl)-1-phenyl-1H-imidazo[4,5-c]pyridin-2(3H)-one (1a)

Compound **1a** was synthesised from **3a** according to general procedure **A** and obtained as brown solid (45 mg, 13% yield for last two steps). ^1^H NMR (400 MHz, DMSO-d_6_) δ 7.78 (d, *J* = 5.5 Hz, 1H), 7.68 − 7.62 (m, 2H), 7.61 (d, *J* = 1.9 Hz, 1H), 7.60 − 7.53 (m, 5H), 7.53 − 7.46 (m, 1H), 6.48 (d, *J* = 5.5 Hz, 1H), 4.96 (s, 2H). ^13 ^C NMR (101 MHz, DMSO-d_6_) δ 152.17, 144.20, 142.05, 136.38, 134.23, 133.47, 133.31, 130.18, 129.95, 129.47, 128.58, 126.71, 109.99, 96.76. ESI-MS: *m/z* 336.77 [M + H]^+^.

##### 4-amino-3-(4-chlorophenyl)-1-(m-tolyl)-1H-imidazo[4,5-c]pyridin-2(3H)-one (1b)

Compound **1b** was synthesised from **3b** according to general procedure **A** and obtained as brown solid (37 mg, 14% yield for last two steps). ^1^H NMR (400 MHz, DMSO-d_6_) δ 7.78 (d, *J* = 5.5 Hz, 1H), 7.68 − 7.62 (m, 2H), 7.58 − 7.52 (m, 2H), 7.48 (t, *J* = 7.7 Hz, 1H), 7.40 − 7.27 (m, 3H), 6.48 (d, *J* = 5.5 Hz, 1H), 4.95 (s, 2H), 2.40 (s, 3H). ^13 ^C NMR (101 MHz, DMSO-d_6_) δ 152.18, 144.17, 142.03, 139.57, 136.45, 134.14, 133.48, 133.29, 130.16, 129.73, 129.46, 129.26, 127.17, 123.75, 109.95, 96.82, 21.27. ESI-MS: *m/z* 350.69 [M + H]^+^.

##### 4-amino-1-(3-bromophenyl)-3-(4-chlorophenyl)-1H-imidazo[4,5-c]pyridin-2(3H)-one (1c)

Compound **1c** was synthesised from **3c** according to general procedure **A** and obtained as brown solid (56 mg, 13% yield for last two steps). ^1^H NMR (400 MHz, DMSO-d_6_) δ 7.84 (t, *J* = 1.9 Hz, 1H), 7.80 (d, *J* = 5.5 Hz, 1H), 7.71 (dt, *J* = 7.8, 1.6 Hz, 1H), 7.67 − 7.64 (m, 2H), 7.62 − 7.58 (m, 1H), 7.56 (d, *J* = 4.3 Hz, 2H), 7.54 (s, 1H), 6.52 (d, *J* = 5.5 Hz, 1H), 4.98 (s, 2H). ^13 ^C NMR (101 MHz, DMSO-d_6_) δ 152.02, 144.25, 142.15, 136.06, 135.72, 133.38, 131.82, 131.50, 130.17, 129.64, 129.50, 125.81, 122.20, 110.04, 99.91, 96.72. ESI-MS: *m/z* 415.38 [M + H]^+^.

##### 4-amino-3-(4-chlorophenyl)-1-cyclohexyl-1H-imidazo[4,5-c]pyridin-2(3H)-one (1e)

Compound **1e** was synthesised from **3e** according to general procedure **A** and obtained as brown solid (24 mg, 13% yield for last two steps). ^1^H NMR (400 MHz, DMSO-d_6_) δ 7.77 (d, *J* = 5.7 Hz, 1H), 7.61 (d, *J* = 8.3 Hz, 2H), 7.45 (d, *J* = 8.4 Hz, 2H), 6.91 (d, *J* = 5.5 Hz, 1H), 4.83 (s, 2H), 4.18 (tt, *J* = 12.3, 3.9 Hz, 1H), 2.11 (qd, *J* = 12.6, 3.4 Hz, 2H), 1.79 (ddd, *J* = 52.3, 37.4, 17.2 Hz, 5H), 1.48 − 1.22 (m, 3H). ^13 ^C NMR (101 MHz, DMSO-d_6_) δ 152.44, 144.02, 141.42, 135.67, 133.61, 133.00, 130.05, 129.31, 109.72, 97.73, 53.26, 29.84, 25.81, 25.08. ESI-MS: *m/z* 343.85 [M + H]^+^.

##### 4-amino-1-(tert-butyl)-3-(4-chlorophenyl)-1H-imidazo[4,5-c]pyridin-2(3H)-one (1f)

Compound **1f** was synthesised from **3f** according to general procedure **A** and obtained as brown solid (32 mg, 15% yield for last two steps). ^1^H NMR (400 MHz, DMSO-d_6_) δ 7.70 (d, *J* = 5.9 Hz, 1H), 7.60 (d, *J* = 8.1 Hz, 2H), 7.43 (d, *J* = 8.2 Hz, 2H), 6.98 (d, *J* = 5.9 Hz, 1H), 4.73 (s, 2H), 1.72 (s, 9H). ^13 ^C NMR (101 MHz, DMSO-d_6_) δ 152.86, 144.09, 140.93, 136.17, 133.67, 133.12, 130.44, 129.23, 110.24, 100.12, 58.95, 29.26. ESI-MS: *m/z* 317.06 [M + H]^+^.

##### 4-amino-3-(4-chlorophenyl)-1-isobutyl-1H-imidazo[4,5-c]pyridin-2(3H)-one (1g)

Compound **1 g** was synthesised from **3g** according to general procedure **A** and obtained as brown solid (45 mg, 10% yield for last two steps). ^1^H NMR (400 MHz, DMSO-d_6_) δ 7.79 (d, *J* = 5.5 Hz, 1H), 7.61 (d, *J* = 8.5 Hz, 2H), 7.46 (d, *J* = 8.6 Hz, 2H), 6.77 (d, *J* = 5.5 Hz, 1H), 4.87 (s, 2H), 3.65 (d, *J* = 7.4 Hz, 2H), 2.12 (dq, *J* = 13.8, 6.9 Hz, 1H), 0.92 (d, *J* = 6.7 Hz, 6H). ^13 ^C NMR (101 MHz, DMSO-d_6_) δ 153.29, 143.82, 141.67, 137.01, 133.66, 132.96, 129.93, 129.35, 122.36, 109.59, 97.01, 48.58, 27.86, 20.21. ESI-MS: *m/z* 317.83 [M + H]^+^.

##### 4-amino-3-(4-chlorophenyl)-1-(2-(dimethylamino)ethyl)-1H-imidazo[4,5-c]pyridin-2(3H)-one (1h)

Compound **1h** was synthesised from **3h** according to general procedure **A** and obtained as brown solid (20 mg, 15% yield for last two steps). ^1^H NMR (400 MHz, DMSO-d_6_) δ 7.79 (d, *J* = 5.5 Hz, 1H), 7.61 (d, *J* = 8.2 Hz, 2H), 7.43 (d, *J* = 8.3 Hz, 2H), 6.77 (d, *J* = 5.5 Hz, 1H), 4.87 (s, 2H), 3.92 (t, *J* = 5.9 Hz, 2H), 2.56 (t, *J* = 5.9 Hz, 2H), 2.20 (s, 6H). ^13 ^C NMR (101 MHz, DMSO-d_6_) δ 153.01, 143.86, 141.74, 136.77, 133.59, 132.86, 129.79, 129.32, 96.88, 56.91, 45.63. ESI-MS: *m/z* 332.93 [M + H]^+^.

##### 4-amino-3-(4-chlorophenyl)-1-(2-methoxyethyl)-1H-imidazo[4,5-c]pyridin-2(3H)-one (1i)

Compound **1i** was synthesised from **3i** according to general procedure **A** and obtained as brown solid (32 mg, 12% yield for last two steps).^1^H NMR (400 MHz, Chloroform-d) δ 7.94 − 7.84 (m, 1H), 7.53 (d, *J* = 8.2 Hz, 2H), 7.42 (d, *J* = 8.2 Hz, 2H), 6.71 (d, *J* = 5.6 Hz, 1H), 4.17 (s, 2H), 4.08 (s, 2H), 3.73 (s, 2H), 3.37 (s, 3H). ^13 ^C NMR (101 MHz, Chloroform-d) δ 153.10, 142.40, 141.29, 137.49, 134.56, 132.63, 129.53, 128.88, 110.08, 97.67, 70.29, 58.94, 41.96. ESI-MS: *m/z* 319.91 [M + H]^+^.

##### 4-amino-3-(4-chlorophenyl)-1-(2-morpholinoethyl)-1H-imidazo[4,5-c]pyridin-2(3H)-one (1j)

Compound **1j** was synthesised from **3j** according to general procedure **A** and obtained as brown solid (40 mg, 15% yield for last two steps). ^1^H NMR (400 MHz, DMSO-d_6_) δ 7.79 (d, *J* = 5.5 Hz, 1H), 7.62 (d, *J* = 8.7 Hz, 2H), 7.43 (d, *J* = 8.7 Hz, 2H), 6.78 (d, *J* = 5.4 Hz, 1H), 4.87 (s, 2H), 3.95 (t, *J* = 6.5 Hz, 2H), 3.56 − 3.44 (m, 4H), 2.60 (t, *J* = 6.5 Hz, 2H), 2.44 (d, *J* = 4.7 Hz, 4H). ^13 ^C NMR (101 MHz, DMSO-d_6_) δ 153.10, 143.83, 141.69, 136.75, 133.62, 132.89, 129.80, 129.35, 109.63, 96.97, 66.62, 56.05, 53.62, 38.78. ESI-MS: *m/z* 374.71 [M + H]^+^.

#### Representative procedure B for the synthesis of compounds 1k–s

##### 4-amino-1-cyclopentyl-3-(4-phenoxyphenyl)-1,3-dihydro-2H-imidazo[4,5-c]pyridin-2-one (1s)

To a solution of **7d** (300 mg, 1.4 mmol) in dichloromethane (10 ml) was added **8 s** (589 mg, 2.8 mmol), Cu(OAc)_2_ (250 mg, 1.4 mmol), pyridine (217 mg, 2.8 mmol), and 4 A molecular sieves (400 mg). The resulting solution was stirred at room temperature under ambient-pressure oxygen atmosphere. After 24 h, the mixture was filtered using ethyl acetate and the solvent was removed under vacuum, then purified by flash chromatography eluting with 20–70% ethyl acetate in petroleum ether to provide the desired product **1s** as brown solid (85 mg, yield: 16%). ^1^H NMR (400 MHz, DMSO-d_6_) δ 7.76 (d, *J* = 5.6 Hz, 1H), 7.46 (t, *J* = 7.9 Hz, 4H), 7.22 (t, *J* = 7.5 Hz, 1H), 7.15 (t, *J* = 8.0 Hz, 4H), 6.75 (d, *J* = 5.5 Hz, 1H), 4.81 (s, 2H), 4.75 (d, *J* = 8.9 Hz, 1H), 2.16 − 1.78 (m, 6H), 1.78 − 1.54 (m, 2H). ^13 ^C NMR (101 MHz, DMSO-d_6_) δ 157.20, 156.38, 152.73, 143.97, 141.24, 135.16, 130.62, 130.28, 129.66, 124.54, 119.75, 118.76, 110.13, 97.28, 53.78, 28.99, 24.94. ESI-MS: *m/z* 387.99 [M + H]^+^.

##### 4-amino-1-cyclopentyl-3-(p-tolyl)-1,3-dihydro-2H-imidazo[4,5-c]pyridin-2-one (1k)

Compound **1k** was synthesised from **7d** (300 mg, 1.4 mmol) and **8k** (374 mg, 2.8 mmol) according to general procedure **B** and obtained as brown solid (68 mg, yield: 16%). ^1^H NMR (400 MHz, DMSO-d_6_) δ 7.75 (d, *J* = 5.6 Hz, 1H), 7.36 (q, *J* = 8.2 Hz, 4H), 6.74 (d, *J* = 5.5 Hz, 1H), 4.80 − 4.73 (m, 1H), 4.70 (s, 2H), 2.41 (s, 3H), 2.14 − 1.83 (m, 6H), 1.75 − 1.56 (m, 2H). ^13 ^C NMR (101 MHz, DMSO-d_6_) δ 152.64, 143.89, 141.14, 138.62, 135.08, 132.27, 130.04, 128.44, 110.19, 97.31, 53.73, 28.98, 24.92, 21.14. ESI-MS: *m/z* 309.90 [M + H]^+^.

##### 4-amino-1-cyclopentyl-3-(4-methoxyphenyl)-1,3-dihydro-2H-imidazo[4,5-c]pyridin-2-one (1l)

Compound **1 l** was synthesised from **7d** (300 mg, 1.4 mmol) and **8 l** (418 mg, 2.8 mmol) according to general procedure **B** and obtained as brown solid (76 mg, yield: 17%). ^1^H NMR (400 MHz, DMSO-d_6_) δ 7.74 (d, *J* = 5.5 Hz, 1H), 7.39 (d, *J* = 8.8 Hz, 2H), 7.10 (d, *J* = 8.8 Hz, 2H), 6.73 (d, *J* = 5.6 Hz, 1H), 4.76 (q, *J* = 8.7 Hz, 1H), 4.69 (s, 2H), 3.89 − 3.77 (m, 3H), 2.12 − 1.82 (m, 6H), 1.76 − 1.58 (m, 2H). ^13 ^C NMR (101 MHz, DMSO-d_6_) δ 159.60, 152.80, 143.91, 141.01, 134.89, 130.05, 127.38, 114.74, 110.43, 97.31, 55.88, 53.71, 28.99, 24.92. ESI-MS: *m/z* 325.96 [M + H]^+^.

##### 4-amino-1-cyclopentyl-3-(4-(trifluoromethyl)phenyl)-1,3-dihydro-2H-imidazo[4,5-c]pyridin-2-one (1m)

Compound **1m** was synthesised from **7d** (300 mg, 1.4 mmol) and **8 m** (523 mg, 2.8 mmol) according to general procedure **B** and obtained as brown solid (75 mg, yield: 15%). ^1^H NMR (400 MHz, DMSO-d_6_) δ 7.91 (d, *J* = 8.2 Hz, 2H), 7.83 (d, *J* = 5.5 Hz, 1H), 7.62 (d, *J* = 8.2 Hz, 2H), 6.79 (d, *J* = 5.5 Hz, 1H), 4.95 (s, 2H), 4.77 (p, *J* = 8.8 Hz, 1H), 2.13 − 1.84 (m, 6H), 1.68 (q, *J* = 8.7, 6.2 Hz, 2H). ^13 ^C NMR (101 MHz, DMSO-d_6_) δ 152.44, 144.27, 141.97, 138.07, 135.99, 128.50, 126.21, 126.17, 125.86, 109.22, 97.20, 53.92, 28.94, 24.94 . ESI-MS: *m/z* 363.93 [M + H]^+^.

##### 4-(4-amino-1-cyclopentyl-2-oxo-1,2-dihydro-3H-imidazo[4,5-c]pyridin-3-yl)benzonitrile (1n)

Compound **1n** was synthesised from **7d** (300 mg, 1.4 mmol) and **8n** (405 mg, 2.8 mmol) according to general procedure **B** and obtained as brown solid (70 mg, yield: 16%). ^1^HNMR (400 MHz, DMSO-d_6_) δ 8.01 (d, *J* = 8.3 Hz, 2H), 7.83 (d, *J* = 5.5 Hz, 1H), 7.57 (d, *J* = 8.3 Hz, 2H), 6.78 (d, *J* = 5.5 Hz, 1H), 5.02 (s, 2H), 4.76 (p, *J* = 8.6 Hz, 1H), 2.12 − 1.83 (m, 6H), 1.73 − 1.60 (m, 2H). ^13 ^C NMR (101 MHz, DMSO-d_6_) δ 152.28, 144.35, 142.17, 138.49, 136.15, 133.15, 128.42, 118.99, 110.26, 108.91, 97.11, 53.94, 28.92, 24.94. ESI-MS: *m/z* 320.76 [M + H]^+^.

##### 4-amino-1-cyclopentyl-3-(4-(hydroxymethyl)phenyl)-1,3-dihydro-2H-imidazo[4,5-c]pyridin-2-one (1o)

Compound **1o** was synthesised from **7d** (300 mg, 1.4 mmol) and **8o** (418 mg, 2.8 mmol) according to general procedure **B** and obtained as brown solid (67 mg, yield: 15%). ^1^H NMR (400 MHz, DMSO-d_6_) δ 7.76 (d, *J* = 5.5 Hz, 1H), 7.50 (d, *J* = 7.8 Hz, 2H), 7.41 (d, *J* = 7.9 Hz, 2H), 6.75 (d, *J* = 5.6 Hz, 1H), 5.35 (t, *J* = 5.9 Hz, 1H), 4.74 (d, *J* = 13.4 Hz, 3H), 4.60 (d, *J* = 5.6 Hz, 2H), 1.99 (ddd, *J* = 40.1, 20.5, 10.5 Hz, 6H), 1.75 − 1.55 (m, 2H). ^13 ^C NMR (101 MHz, DMSO-d_6_) δ 152.64, 143.88, 143.55, 141.16, 135.14, 133.24, 128.31, 127.36, 110.15, 97.32, 62.79, 53.76, 28.99, 24.93. ESI-MS: *m/z* 325.74 [M + H]^+^.

##### 4-amino-1-cyclopentyl-3-(3-fluorophenyl)-1,3-dihydro-2H-imidazo[4,5-c]pyridin-2-one (1p)

Compound **1p** was synthesised from **7d** (300 mg, 1.4 mmol) and **8p** (385 mg, 2.8 mmol) according to general procedure **B** and obtained as brown solid (77 mg, yield: 18%). ^1^H NMR (400 MHz, DMSO-d_6_) δ 7.79 (d, *J* = 5.5 Hz, 1H), 7.67 − 7.52 (m, 1H), 7.45 − 7.31 (m, 2H), 7.28 (d, *J* = 7.9 Hz, 1H), 6.77 (d, *J* = 5.6 Hz, 1H), 4.85 (s, 2H), 4.79 − 4.69 (m, 1H), 2.11 − 1.84 (m, 6H), 1.74 − 1.59 (m, 2H). ^13 ^C NMR (101 MHz, DMSO-d_6_) δ 152.44, 144.05, 141.62, 136.13, 135.54, 130.89, 124.46, 116.00, 115.76, 115.71, 115.51, 109.69, 97.25, 53.84, 28.97, 24.95. ESI-MS: *m/z* 313.83 [M + H]^+^.

##### 4-amino-3-(benzo[d][1,3]dioxol-5-yl)-1-cyclopentyl-1H-imidazo[4,5-c]pyridin-2(3H)-one (1q)

Compound **1q** was synthesised from **7d** (300 mg, 1.4 mmol) and **8q** (457 mg, 2.8 mmol) according to general procedure **B** and obtained as brown solid (88 mg, yield: 19%). ^1^H NMR (400 MHz, DMSO-d_6_) δ 7.74 (d, *J* = 5.4 Hz, 1H), 7.17 − 7.00 (m, 2H), 6.91 (d, *J* = 8.3 Hz, 1H), 6.72 (d, *J* = 5.6 Hz, 1H), 6.16 (s, 2H), 4.79 (s, 2H), 4.73 (d, *J* = 8.9 Hz, 1H), 2.12 − 1.81 (m, 6H), 1.76 − 1.58 (m, 2H). ^13 ^C NMR (101 MHz, DMSO-d_6_) δ 152.75, 148.00, 147.90, 143.92, 141.07, 134.87, 128.41, 122.37, 110.42, 109.93, 108.39, 102.40, 97.25, 53.70, 28.98, 24.92. ESI-MS: *m/z* 339.74 [M + H]^+^.

##### 4-amino-1-cyclopentyl-3-(1H-indol-5-yl)-1,3-dihydro-2H-imidazo[4,5-c]pyridin-2-one (1r)

Compound **1r** was synthesised from **7d** (300 mg, 1.4 mmol) and **8r** (443 mg, 2.8 mmol) according to general procedure **B** and obtained as brown solid (78 mg, yield: 17%). ^1^H NMR (400 MHz, DMSO-d_6_) δ 11.43 (s, 1H), 7.72 (d, *J* = 5.5 Hz, 1H), 7.65 (d, *J* = 1.9 Hz, 1H), 7.54 (d, *J* = 8.5 Hz, 1H), 7.51 (t, *J* = 2.8 Hz, 1H), 7.13 (dd, *J* = 8.5, 2.1 Hz, 1H), 6.74 (d, *J* = 5.6 Hz, 1H), 6.54 (t, *J* = 2.4 Hz, 1H), 4.77 (p, *J* = 8.7 Hz, 1H), 4.61 (s, 2H), 2.12 − 1.85 (m, 6H), 1.75 − 1.57 (m, 2H). ^13 ^C NMR (101 MHz, DMSO-d_6_) δ 153.12, 143.99, 140.78, 135.87, 134.64, 127.97, 127.74, 126.28, 121.63, 120.66, 112.19, 110.89, 102.03, 97.27, 53.68, 29.03, 24.93. ESI-MS: *m/z* 334.96 [M + H]^+^.

### Enzymatic assay

According to the manufacturer’s instructions, single point inhibition rate at 1 µM and IC_50_ determinations of compounds against Src and Fyn (Carna Biosciences, Kobe, Japan, Product Number: 08–173 for Src. Product Number: 08–168 for Fyn) were performed with the HTRF^®^ KinEASETM-TK assay from Cisbio. Positive drug PP2 was purchased from Bidepharm and other compounds was synthesised. HTRF^®^ KinEASETM-TK assay involves enzymatic step and detection step. During the enzymatic step, kinase buffer containing 5 mM DTT, 1 mM MgCl_2_, 5 µM protein substrate, a certain concentration of ATP, compounds with different concentrations, was added to 384 plates. The Src kinase reaction was started by the addition of 0.111 ng/µL Src at 37 °C, The Src kinase reaction was started by the addition of 0.333 ng/µL Fyn at 37 °C. ATP concentrations were set at their respective Km values (16 µM for Src and 51.76 µM for Fyn). After the reaction was completed (reaction times: 40 min for Src and Fyn), the reaction was stopped during the detection step by the addition of detection reagents which contain TK-Antibody labelled with Eu3+-Cryptate, streptavidin-XL665 and EDTA. Then, the TR-FRET signal was measured after an additional incubation for 1 h to quantify the phosphorylation of the substrate. The fluorescence is measured at 620 and 665 nM using Infinite M1000 Pro plate reader (Tecan I-control). Then GraphPad prism version 7 (GraphPad Software, Inc., La Jolla, CA) was used to analyse the ratio of fluorescence intensity 665/620 under nine different inhibitor concentrations, and IC_50_ was calculated. Two duplicate wells were set for each reaction, each IC_50_ was measured at nine different concentrations in the three-fold dilution step starting at 1 µM and determined at least two times.

### Cell proliferation inhibition assay

GBM cell lines U87, U251, and T98G (purchased from the American Type Culture Collection Company, Manassas, VA) were cultured in Roswell Park Memorial Institute (RPMI) 1640 medium containing 10% foetal bovine serum (FBS) at 37 °C in 5% CO_2_ incubator. The U87-EGFRvIII cell line established with EGFRvIII, kindly provided by Professor Xingmei Zhang, and grown in DMEN supplement with 10% FBS and 200 mg/ml G418 (Sigma-Aldrich, St. Louis, MO) at 37 °C in 5% CO_2_ incubator. The anti-proliferative activities of the compounds to be tested against four glioblastoma cell lines were determined by standard MTT method. In order to determine the anti-proliferation effect of the tested compounds, four cell lines were inoculated at a density of 2 × 10^3^ cells/ml and treated with compounds at a concentration of 1–100 µM. The cells were co-incubated with different concentrations of the tested drugs for 72 h, and 10% MTT solution diluted in 100 µL medium was added. After 4 h of incubation, the absorbance value of each hole was measured at 570 nM with an enzyme-linked immunosorbent assay plate reader. All compounds were tested three times in each cell line.

### Molecular modelling

#### Molecular docking

Molecular docking was carried out on the induced fit docking (IFD) module of Schrödinger software package (Maestro version 11.1, New York, NY, USA)^36^ . The crystal structures of Src (PDB ID: 3GEQ)^37^, Fyn (PDB ID: 2DQ7)^38^, Lyn (PDB ID: 2ZV9)^39^, Lck (PDB ID: 1QPE)^40^, and Hck (PDB ID: 3VRY)^41^ were obtained from protein data bank (PDB) (http://www.rcsb.org/). The proteins were imported and prepared in Protein Preparation Wizard module by repairing missing side chains or missing loops, adding hydrogen, removing crystallographic water, and assigning partial charges. Then the proteins were optimised using Optimised Potentials for Liquid Simulations OPLS2005 force field with the default root mean square deviation (RMSD) value of 0.3 Å. Compound **1s** and reference compound PP2 were constructed using Maestro version 11.1 and were predict ionisation and tautomeric states by Ligprep module.

IFD process was referred to previous research. Briefly, the active sites in each protein were generated in the centre of the original ligand under the default parameters. During IFD, the initial Glide docking was applied to generated 20 poses, then all the initial poses were taken into prime refinement to optimise the side chains within 6.0 Å of ligand poses. After that, Extra-precision (XP)^42^ docking was used to redock the ligand into the active site of each receptor. The best scoring conformer of each docking result was sent to molecular dynamic (MD) simulation.

#### Molecular dynamics simulation

MD simulations were performed using the Desmond programme^43^. The TIP3P water model^44^ was used to simulate water molecules in an orthorhombic box under periodic boundary conditions, positioned such that the walls were at minimum 10 Å distance from any system atom. Counter ions (i.e. Na^+^/Cl^−^) were added to balance the system charge. The default Desmond protocol was performed for minimisation and relaxation of the ligand-protein complexes in the NPT ensemble. Periodic boundary conditions and the OPLS2005 force field were applied in the MD simulations. Using Nose–Hoover temperature coupling and isotropic scaling, the temperature and pressure were kept constant at 310 K and 1 atmospheric pressure, respectively. The simulations were run for 20 ns in the NPT ensemble, saving the obtained configurations at 10 ps intervals. Each MD trajectory was sent to simulation interactions diagram for analysis. PyMOL was used to visualise and analyse the structures^45^.

## Results and discussion

### Rational design

**Figure 2.**
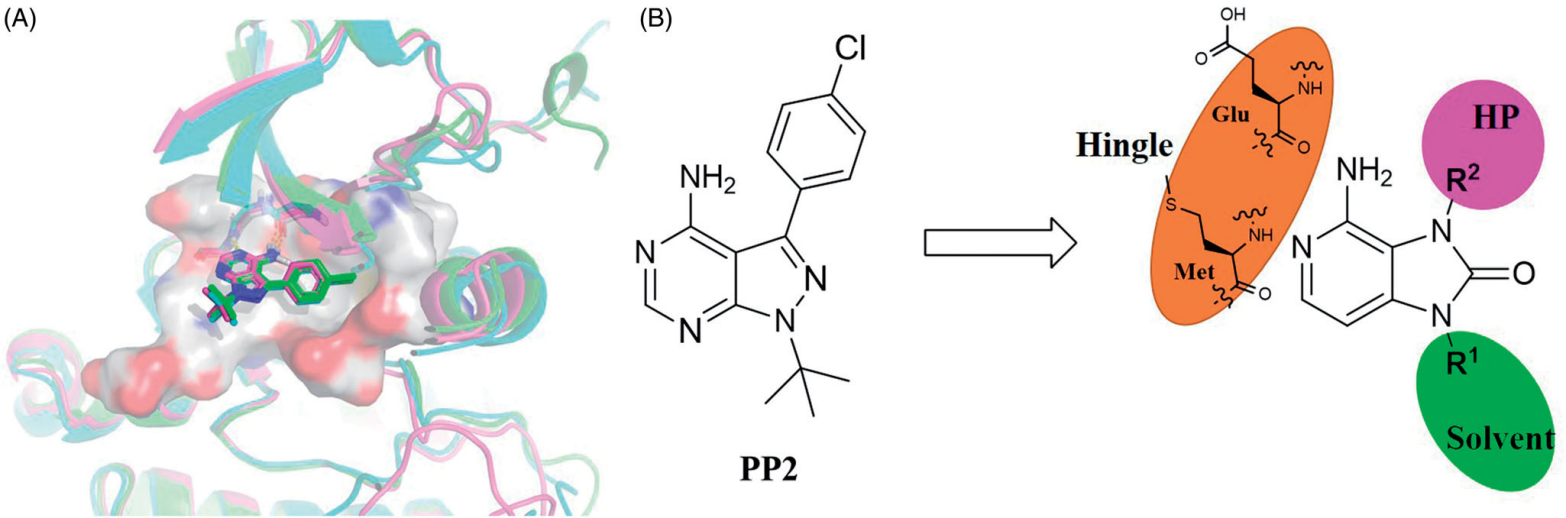
(A) Overlapping model of PP2 bound to Src (PDB ID: 3GEQ), Lyn (PDB ID: 2ZV9), Lck (PDB ID: 1QPE), the crystal structures were displayed as ribbon and initial ligands were exhibited as stick (cyan for Src, purple for Lyn, green for Lck). (B) Design strategy of imidazo[4,5-c]pyridin-2-one compounds.

We envisaged the possibility of further derivation and optimisation of small molecular PP2 to design novel SFK inhibitors with unique binding mode and higher activity. Through the observation of cocrystal structures between PP2 and SFK members that have been reported (Figure 2(A)), we found that PP2 adopted the same binding mode in ATP binding pocket of Src, Lyn, and Lck. In detail, PP2 forms two hydrogen bonds with the hinge region. The amino group on the pyrimidine ring C4 acts as a hydrogen bond donor to form a hydrogen bond with the carbonyl group of glutamic acid. The nitrogen at N5 position acts as hydrogen bond acceptor in the interaction with methionine. The 4-chlorophenyl group on C3 extends inward into hydrophobic pocket, and N1 tert-butyl group is solvent exposed.

Notably, N7 position at pyrimidine ring of PP2 did not form a key interaction with SFKs, so we tried to replace pyrimidine with pyridine. The pyrazole ring of PP2 was replaced by imidazolone ring based on bioisosterism. Therefore, a new hinge region junction, imidazo[4,5-c]pyridin-2-one, was formed. Meanwhile, the derivatives were carried out on the N1 and N3 position of imidazolone ring (Figure 2(B)). Introducing different polar and nonpolar groups on N1 of imidazolone to explore the optimal interaction with the amino acid surrounding of solvent region. Besides, groups with different volumes were introduced into N3 of imidazolone ring to explore the size of hydrophobic pocket, aiming at form the best interaction with surrounding amino acids.

### Chemistry

First, a series of derivatives **1a–j** with different substituents on N1 position were synthesised by retaining 4-chlorophenyl group on the N3 position of imidazolone ring. Cyclopentyl group was subsequently identified as the best substituent by enzymatic assays, then compounds **1h–s** with different substituents on N3 were synthesised by retaining cyclopentyl group on N1 position of imidazolone ring.

**Figure 3.**
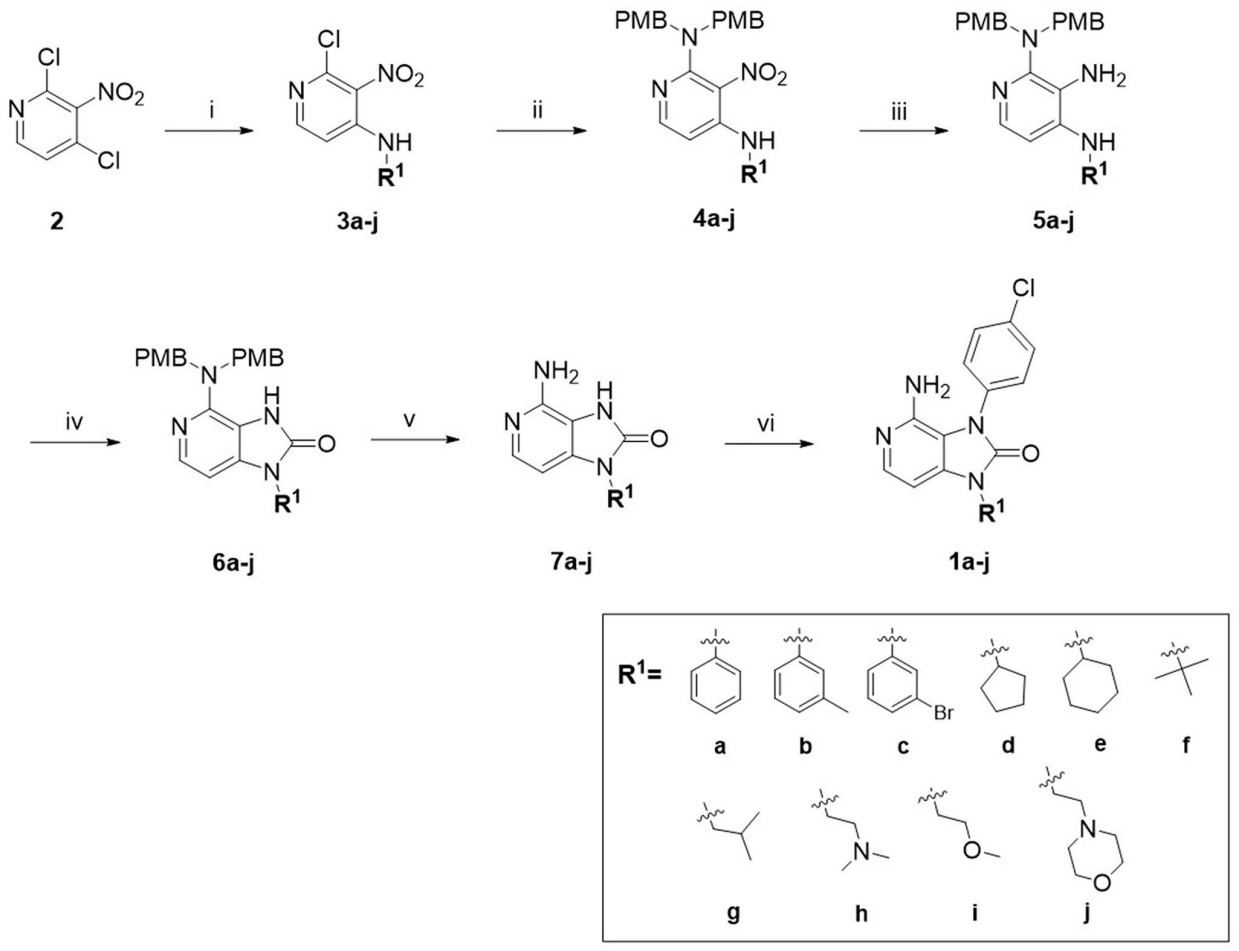
Synthesis of the target compounds **1a–j**. Reagents and conditions: (i) Substituted amines, Et_3_N, DMF, r.t., overnight. (ii) Bis(4-methoxybenzyl)-amine, isopropanol, reflux, overnight. (iii) Fe, AcOH/EtOH/H_2_O, 80 °C, 2 h. (iv) CDI, THF, reflux, overnight. (v) TFA, DCM, r.t., 4 h. (vi) 4-chlorophenylboronic acid, Cu(OAc)_2_, Py, 4 A MS, air, r.t., 24 h.

Compound **1a–j** was prepared according to the synthetic route of Figure 3. First, **1a–j** were prepared starting with the reaction of **2** and various substituted amines in the presence of triethylamine to produce **3a–j**. Subsequent reaction of **3a–j** with bis(4-methoxybenzyl) amine, followed by nitro reduction yielded intermediates **5a–j**. The intermediates **5a–j** underwent a ring closure using 1,1′-carbonyldiimidazole to afford **6a–j**. Then **6a–j** reacted with trifluoroacetic acid for 4 h to remove 4-methoxybenzyl group and expose amino group to generate **7a–j**. Finally, **7a–j** reacted with 4-chlorophenylboronic acid to generate end products **1a–j** through Chan–Lam coupling reaction^46^.

**Figure 4.**
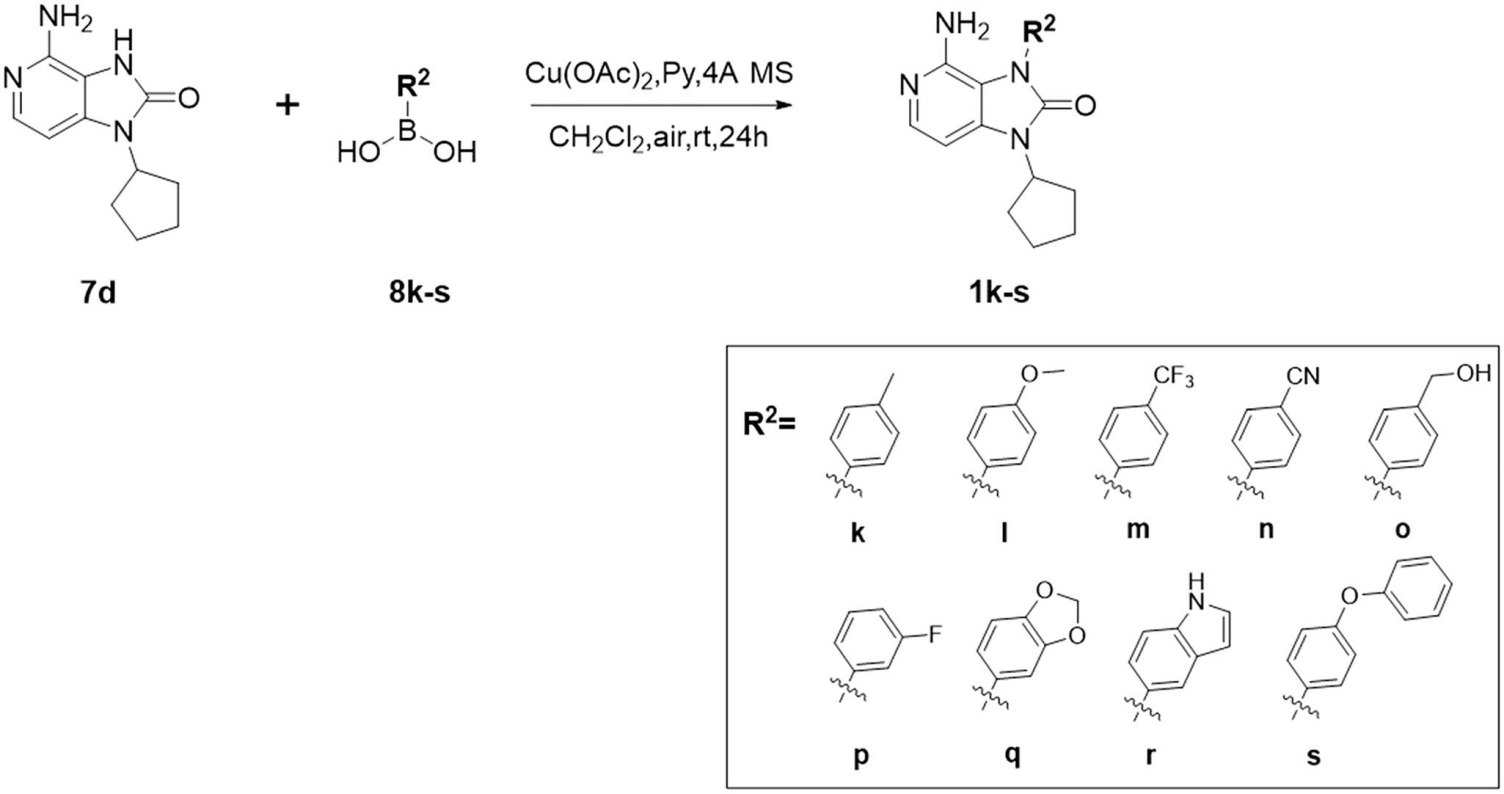
Synthesis of the target compounds **1k–s**.

Compound **1k–s** was prepared according to the synthetic route of Figure 4. Compound **7d** reacted with various substituted phenylboronic acids *via* Chan–Lam C–N coupling reaction with the participation of oxygen, copper acetate, and pyridine to afford end products **1k–s**.

### Kinase inhibitory activity

Since the members of the SFKs have high homology^47^, we selected Src and Fyn, the two most studied and representative SFK members as enzymatic assay targets to screen all synthesised compounds. Small molecular PP2 was used as control drug to detect its inhibitory activity on Src and Fyn. We first synthesised ten imidazo[4,5-c]pyridin-2-one compounds **1a–j**, which are characterised by retaining 4-chlorophenyl group at N3 position and introducing different substitutions at N1 of imidazolone ring. As shown in Table 1, compounds **1d**, **1e,** and **1f**, containing cyclopentyl, cyclohexyl, tert-butyl group at N1, respectively, showed significant inhibitory activity against Src and Fyn at 1 µM concentration. IC_50_ values of **1d**, **1e** were then tested, in particular, compound **1d** exhibited similar kinase inhibitory activity to the positive control drug PP2, with 0.19 and 0.24 µM against Src and Fyn, respectively. However, other compounds showed no obvious inhibition. Compounds **1a**, **1 b**, **1c**, and **1i** exhibited moderate inhibitory effect on Src and low inhibitory activity on Fyn, showing selectivity to a certain extent.

**Table 1.** *In vitro* Src and Fyn inhibitory activity of compounds **1a–j.**

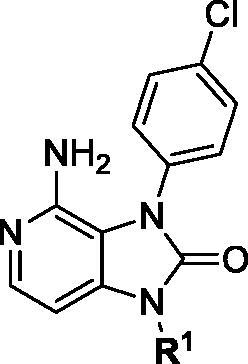							
COM.	R^1^	Inhibition (1 μM, %)^a^		COM.	R^1^	Inhibition (1 μM, %)^a^	
		Src	Fyn			Src	Fyn
**1a**	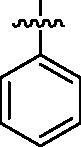	78	35	**1f**	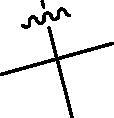	84	62
**1b**	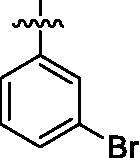	54	11	**1g**	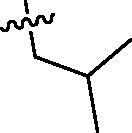	16	19
**1c**	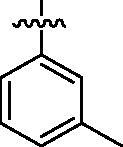	68	31	**1h**	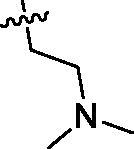	18	19
**1d**	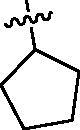	96 (0.19)^b^	90 (0.24)^b^	**1i**	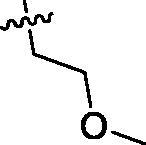	65	24
**1e**	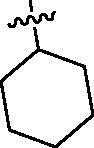	88 (0.073)^b^	76 (0.58)^b^	**1j**	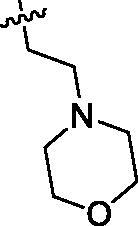	5	12
**PP2**	–	93 (0.16)^b^	95 (0.18)^b^	–	–	–	–

^a^Values indicate the kinase inhibition % at 1 μM, which are the average of two independent experiments.

^b^Values in the parentheses indicate IC_50_ (μM), which are the average of two independent experiments.

Based on the above enzyme activity results, nine analogues **1k–s** were synthesised by optimising compound **1d** to further improve its potency. The R^2^ moiety was differently functionalised and retaining cyclopentyl group at N1 position. As it can be appreciated from Table 2, compounds **1k, 1q,** and **1s** have strong inhibitory effect on Src and Fyn at 1 µM concentration. IC_50_ values of **1q**, **1s** were then tested, compound **1s**, which bearing 4-phenoxyphenyl group on N3, exhibited comparable kinase inhibitory activity to PP2, with IC_50_ values of 0.15 and 0.21 µM against Src and Fyn, respectively.

**Table 2.** In *vitro* Src and Fyn inhibitory activity of compounds **1k–s**.

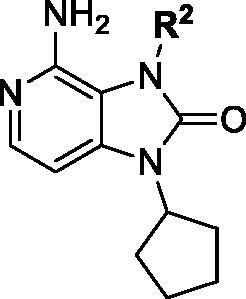							
COM.	R^2^	Inhibition (1 μM, %)^a^		COM.	R^2^	Inhibition (1 μM, %)^a^	
		Src	Fyn			Src	Fyn
**1k**	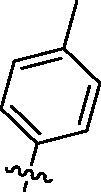	88	65	**1p**	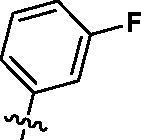	66	19
**1l**	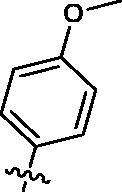	91	28	**1q**	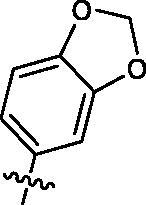	85 (0.22)^b^	74 (0.73)^b^
**1m**	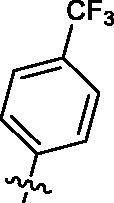	11	−2	**1r**	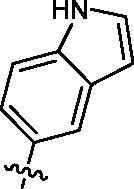	84	30
**1n**	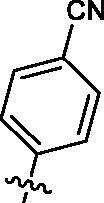	45	44	**1s**	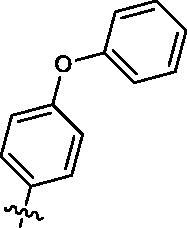	93 (0.15)^b^	90 (0.21)^b^
**1o**	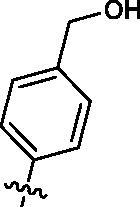	19	20	**PP2**	–	93 (0.16)^b^	95 (0.18)^b^

^a^Values indicate the kinase inhibition % at 1 μM, which are the average of two independent experiments.

^b^Values in the parentheses indicate IC_50_ (μM), which are the average of two independent experiments.

### Preliminary structure-kinase activity relationships

**Figure 5.**
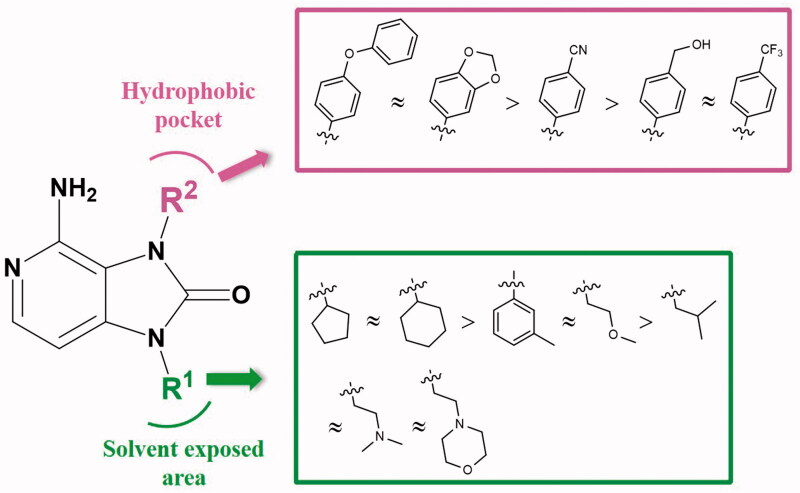
Preliminary structure-activity relationships.

Based on the results of enzyme activity evaluation, we summarised the preliminary structure–activity relationship of the synthesised compounds (Figure 5). In general, when R^2^ group is substituted by 4-chlorophenyl group, the inhibitory activity of aliphatic ring substituent at R^1^ position was better than that of aromatic ring, short chain alkanes and their derivative groups, of which cyclopentyl was the preferred group. When R^1^ group is substituted by cyclopentyl group, the inhibitory activity of larger volume group at R^2^ position, such as 4-phenoxyphenyl, benzodioxol group was better than that of the smaller single substituted phenyl groups, of which 4-phenoxyphenyl was the preferred group.

### *In vitro* anti-proliferative activity

As mentioned above, SFKs are upregulated and hyperactivated in GBM, and their activity regulates proliferation and differentiation of cancer cells. The four most active compounds in terms of enzymatic activities **1d**, **1e**, **1q**, **1s,** and positive compound PP2 were subsequently evaluated for their antiproliferative activities against GBM cell lines (Table 3). Among the four target compounds, **1s** showed effective antiproliferative activity, which was closely related to the IC_50_ values determined by enzyme inhibition assays. The IC_50_ values of **1s** on U87, U251, T98G, and U87-EGFRvIII cells were 30.3, 24.6, 16.4, and 50.5 µM, respectively, showing the similar activity with positive compound PP2.

**Table 3.** Antiproliferative activity of target compounds against GBM cell lines (IC_50_, μM)^a^.

COM.	U87	U251	T98G	U87-EGFRvIII
**1d**	63.28 ± 10.45	33.28 ± 3.36	20.62 ± 0.30	67.19 ± 3.20
**1e**	53.61 ± 2.51	36.1 ± 2.53	20.07 ± 0.67	>100
**1q**	47.04 ± 3.78	30.02 ± 0.93	20.73 ± 0.67	68.98 ± 8.39
**1s**	30.30 ± 0.84	24.61 ± 2.28	16.39 ± 2.12	50.46 ± 0.97
**PP2**	39.94 ± 6.91	21.14 ± 2.16	12.52 ± 2.37	49.11 ± 7.76

^a^IC_50_ values are presented as mean values of at least three independent experiments.

### Molecular simulation

**Figure 6.**
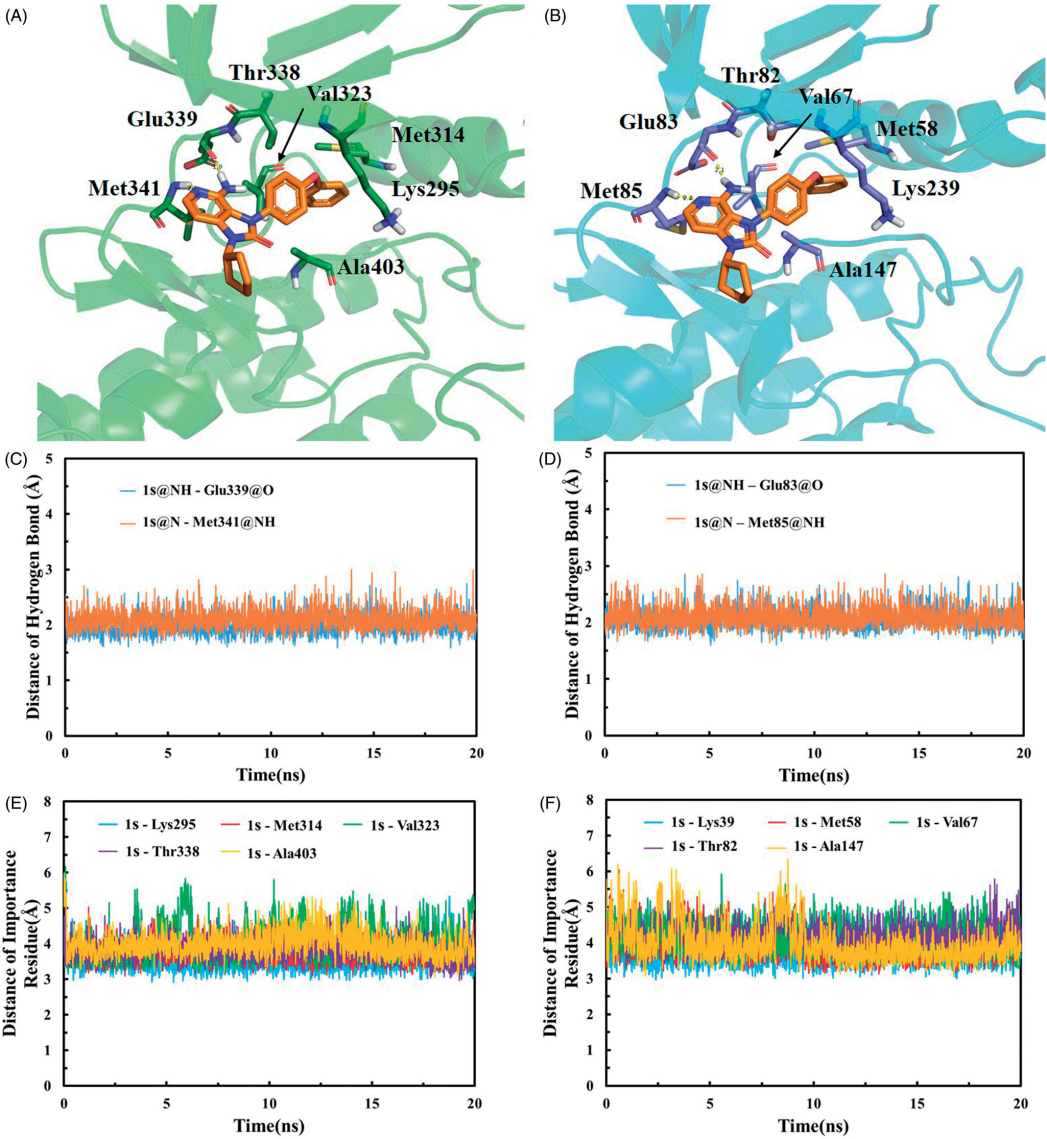
Representative structure of compound **1s** interacted with Src (A) and Fyn (B). The proteins were displayed as ribbon, and the interacted residues were exhibited as stick (green for Src and cyan for Fyn). Compound **1s** was showed as orange stick, and the hydrogen bonds were displayed as yellow dashes. The time-dependent change of hydrogen bond length between compound **1s** and residues in Src (C) and Fyn (D). The time-dependent change of distance between compound **1s** and important residues in Src (E) and Fyn (F).

In order to investigate the binding details of compound **1s** interacted with SFKs, MD simulation was employed on five SFK members Src, Fyn, Lyn, Lck, and Hck, which kinase domain crystal structures have been reported. The RMSD of all MD systems indicated that each system reaches equilibrium and remain stable within 20 ns MD simulation (Figure S1). The representative structures of **1s** are shown in Figure 6(A,B). The aminopyridine group of **1s** formed two hydrogen bonds with the hinge residues of Src (Glu339 and Met341) and Fyn (Glu83 and Met85), and the hydrogen bonds maintain stable in each simulation (Figure 6(C,D)). In addition, the 4-phenoxyphenyl group of compound **1s** reached into the hydrophobic pocket of Src and Fyn, and had hydrophobic interaction with lots of residues: Lys295, Met314, Val323, Thr338, and Ala403 of Src (Lys39, Met58, Val67, Thr82, and Ala147 of Fyn). These hydrophobic interactions also fluctuate stably in the whole time (Figure 6(E,F)). The hydrogen bonds together with hydrophobic interaction facilitated **1s** bind to Src and Fyn.

The cyclopentyl group did not seem to interact with many residues, but stilled play a significant role in **1s** inhibiting against SFKs. The rigid structure of cyclopentyl group could form a stable dihedral angle (about 70 degrees) with imidazolone ring (Figure S2). The stereospecific blockade generated by this dihedral angle might drive **1s** into ATP pocket in a specific direction, which promoted the formation of hydrogen bond and hydrophobic interaction.

The conformation of **1s** binding into the ATP pocket of Lyn, Lck, and Hck showed similar characteristics with Src, including the stable dihedral angle between cyclopentyl group and imidazolone ring, the hydrogen bond with hinge and hydrophobic interaction in hydrophobic pocket (Figures S3 and S4). The common interaction between **1s** and SFKs (Src, Fyn, Lyn, Lck, and Hck) suggested that **1s** may have similar inhibitory activity against Lyn, Lck, and Hck as Src. Furthermore, in *silico* ADME prediction indicated that the physicochemical properties of the compounds **1s** were in line with the main characteristics of CNS drugs and suggested that **1s** may have good CNS permeability (Table S1). Previous studies have been reported that one of the main reasons for the failure of dasatinib treatment in GBM clinical trials is related to physiological disorders, such as blood-brain barrier^32^. The prediction indicates that compound **1s** may have better CNS activity and BBB permeability than dasatinib. It is necessary to carry out related experiments to study the accuracy of these parameters prediction.

Some hints for structural optimisation can be obtained from the MD simulation results. There remained open spaces in the conformation of compound **1s** bound to SFKs, which may accommodate new substituents to improve the inhibitory activity (Figure S5). One of these spaces located between the cyclopentyl of **1s** and Asp348 on Src (also exists in the corresponding position on other SFKs), which forms hydrogen bonds with the ribose moiety of ATP. The cyclopentyl of **1s** could be modified with amine moieties aiming to contribute salt bridge or hydrogen bond with Asp384. Another available space extends towards hydrophobic pocket. The phenyl group of **1s** could be replaced by a smaller substituent (or not) ring, helping the compound slip into hydrophobic pocket with less steric clash.

Due to the particular location of GBM in brain, targeting this tumour is a complicated problem. The BBB avoids the exogenous insults and the outflow of circulating substances in the brain, but its tightly connected structure and the active efflux mediated by ATP-binding cassettes (ABC) transporters on the barrier, such as P-glycoprotein (P-gp) are enemies of SFK inhibitors for GBM treatment. Multidrug resistance caused by overexpression of ABC transporters in the BBB makes it difficult to achieve effective drug concentrations in tumours. Therefore, targeting transporters could be a valuable approach that improves drug delivery to tumour sites^48^.

On the basis of the above study, it is necessary to investigate whether our best compound is a substrate or an inhibitor of a transporter, and thus to speculate its possible adverse reactions or related drug-drug interactions. In addition, there is a possibility to enhance inhibitor efficacy by structurally modifying or prodrug modifying our optimal compound to become a substrate of uptake transporters or to avoid excretion by efflux transporters, thereby increasing drug uptake and concentrating in target organs.

## Conclusion

Because of the crucial role in GBM pathogenesis, SFKs could be considered as interesting targets for therapeutic intervention. Based on structural characteristics of SFK inhibitor PP2, a series of imidazo[4,5-c]pyridin-2-one derivatives were designed and synthesised as novel SFK inhibitors. After two rounds of structural optimisation at N1 and N3 of imidazolone ring, compound **1s** with potential kinase inhibition was screened out, with IC_50_ values were 0.15 and 0.21 µM against Src and Fyn, respectively. **1s** also showed effective antiproliferative activity against GBM cell lines *in vitro*, with IC_50_ values of 30.3, 24.6, 16.4, and 50.5 µM on U87, U251, T98G, and U87-EGFRvIII cells, which were comparable to that of PP2. Furthermore, MD simulations disclosed the binding model of **1s** at the ATP-binding sites of SFKs. ADME prediction revealed that **1s** meet the criteria for CNS-acting drugs. These findings indicated that we have developed a novel SFK inhibitor, which provides a basis for further study of imidazole[4,5-c]pyridine-2-one derivatives to fully understand its potential as an effective anti-GBM drug. Further studies to enhance the potential activity and druggability of imidazole[4,5-c]pyridine-2-one derivatives based on **1s** are in progress.

## Supplementary Material

Supplemental Material
