# Supplementary material for: Identification of imidazo[4,5-c]pyridin-2-one derivatives as novel Src family kinase inhibitors against glioblastoma
Source: J Enzyme Inhib Med Chem. 2021 Jul 8;36(1):1541–52. doi: 10.1080/14756366.2021.1948542 (PMC8274516; doi:10.1080/14756366.2021.1948542)
Supplement: Supplemental Material [file IENZ_A_1948542_SM6417.pdf]

Identification of imidazo[4,5-c]pyridin-2-one derivatives as novel Src family kinase inhibitors against Glioblastoma

Lishun Zhang[a, 1], Zichao Yang[a, 1], Huiting Sang[a], Ying Jiang[a], Mingfeng Zhou[a], Chuan Huang[a], Chunhui Huang[a], Xiaoyun Wu[a], Tingting Zhang[a, b], Xingmei Zhang[c], Shanhe Wan[a]* and Jiajie Zhang[a]*

[a]*Guangdong Provincial Key Laboratory of New Drug Screening, School of Pharmaceutical Science, Southern Medical University, Guangzhou, China;* [b]*Guangdong Provincial Key Laboratory of New Drug Design and Evaluation, Guangzhou, China;* [c]*Guangdong Province Key Laboratory of Psychiatric Disorders, Department of Neurobiology, School of Basic Medical Sciences, Southern Medical University, Guangzhou, China*

[1] These authors contributed equally to this work.

Address for correspondence: Jiajie Zhang, zhangjj@smu.edu.cn; Shanhe Wan, wansh@smu.edu.cn. Guangdong Provincial Key Laboratory of New Drug Screening, School of Pharmaceutical Sciences, Southern Medical University, Guangzhou, 510515, China

Table of Content

NMR Spectra data for compounds **1a-s** Pages 2-20

Molecular modeling Pages 21-24

NMR Spectra data for compound 1a-s

^{1}H NMR Spectrum of Compound **1a**

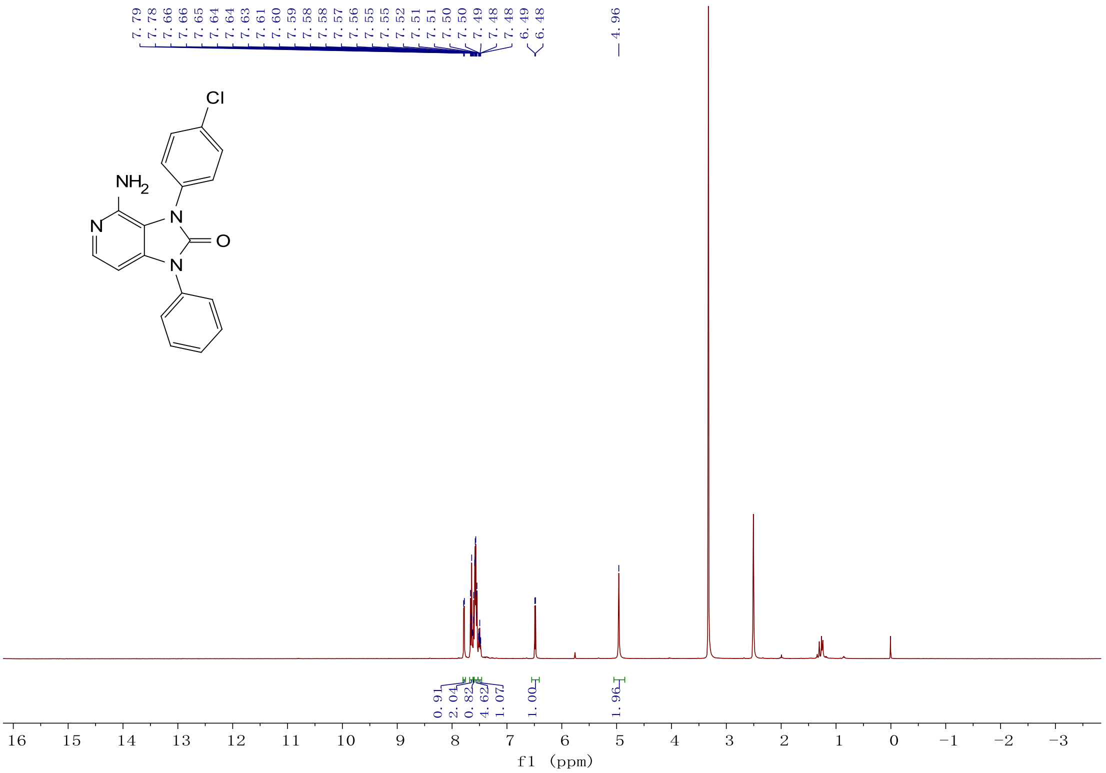

^{13}C NMR Spectrum of Compound **1a**

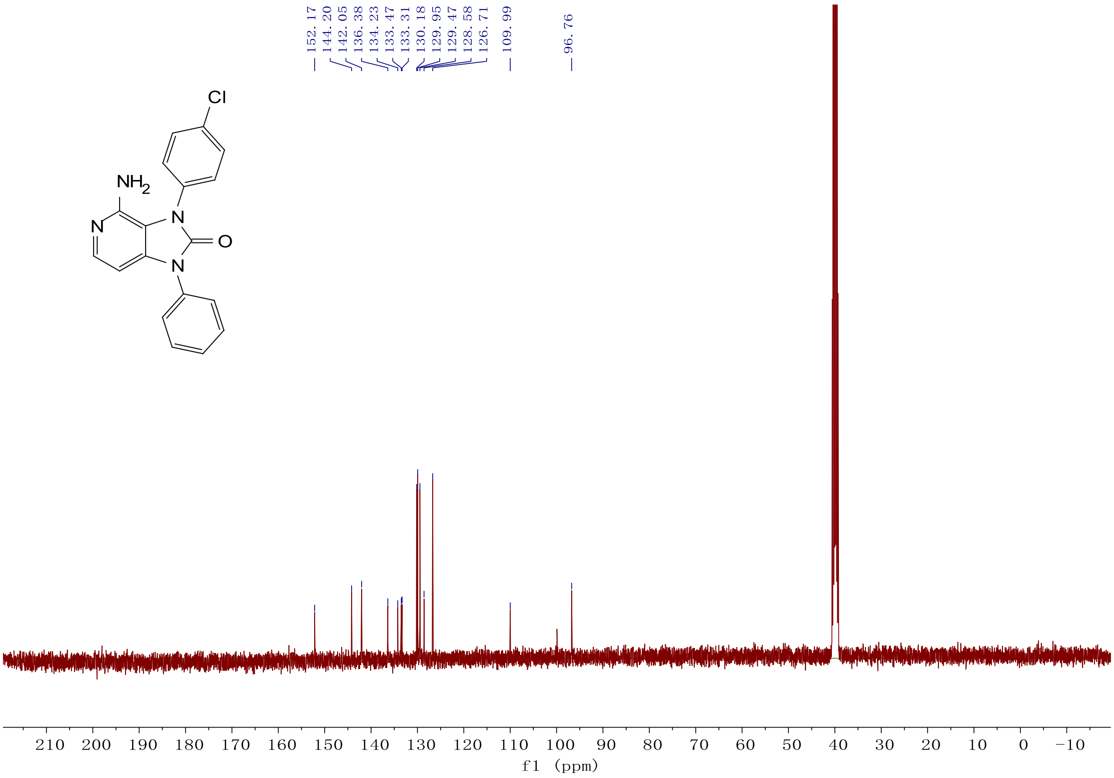

^{1}H NMR Spectrum of Compound **1b**

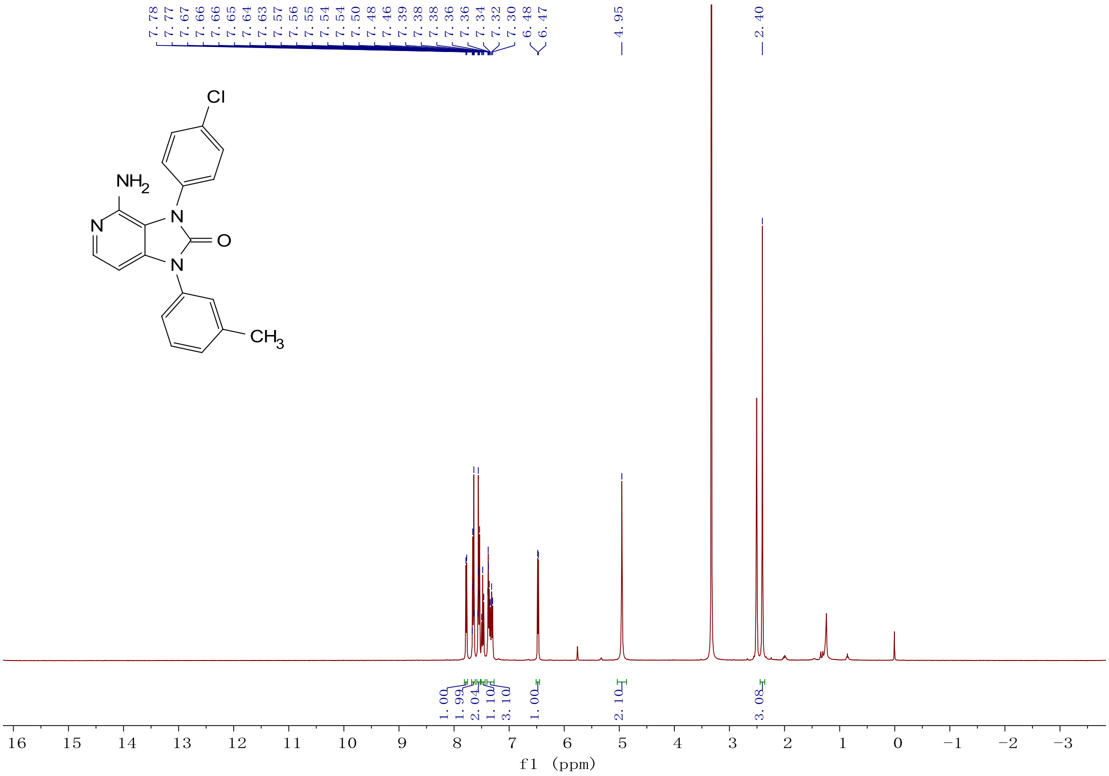

^{13}C NMR Spectrum of Compound **1b**

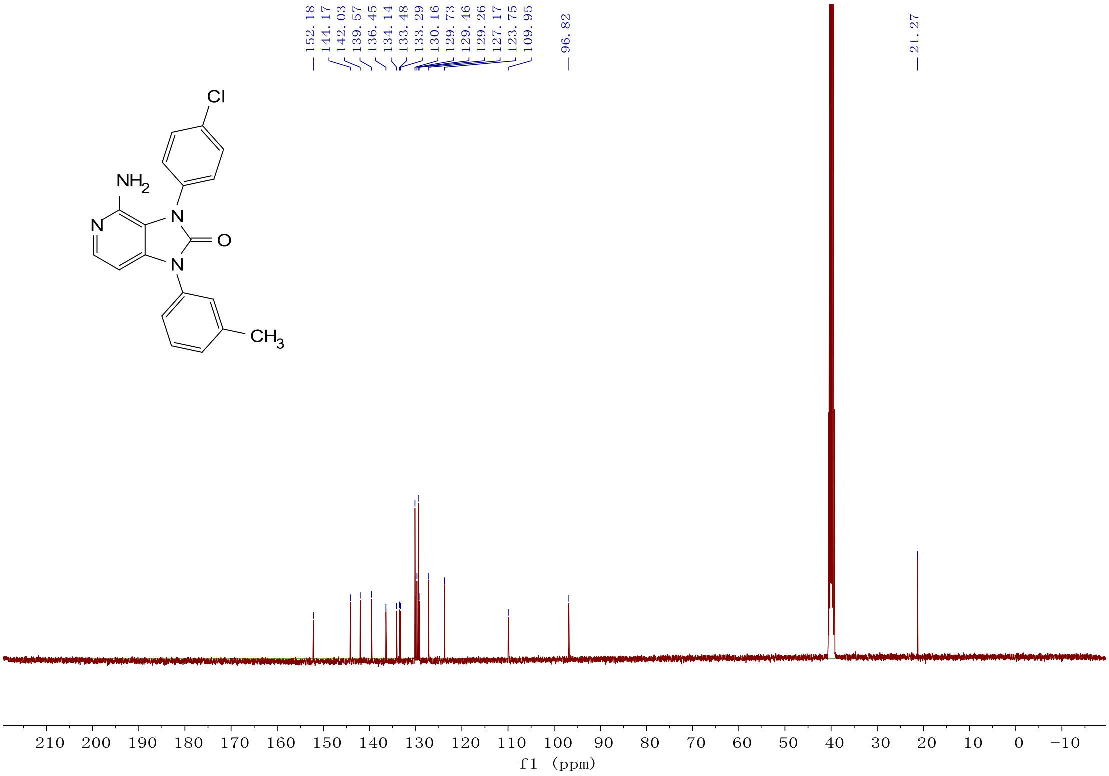

^{1}H NMR Spectrum of Compound **1c**

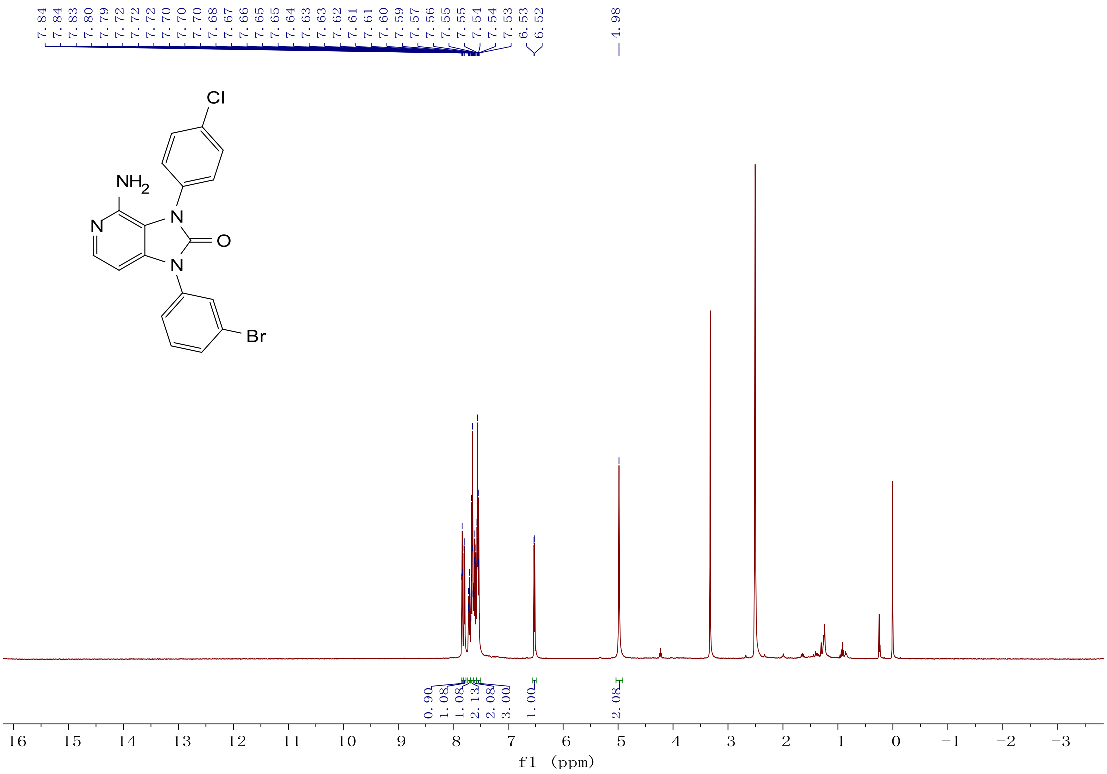

^{13}C NMR Spectrum of Compound **1c**

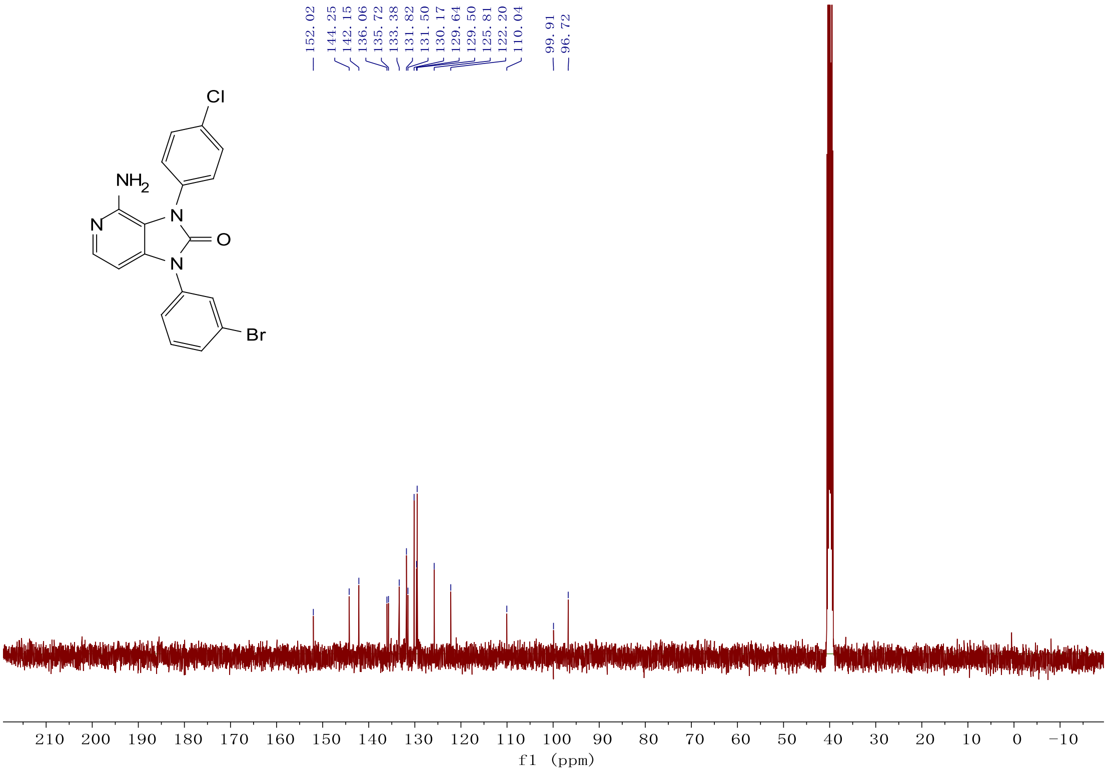

^{1}H NMR Spectrum of Compound **1d**

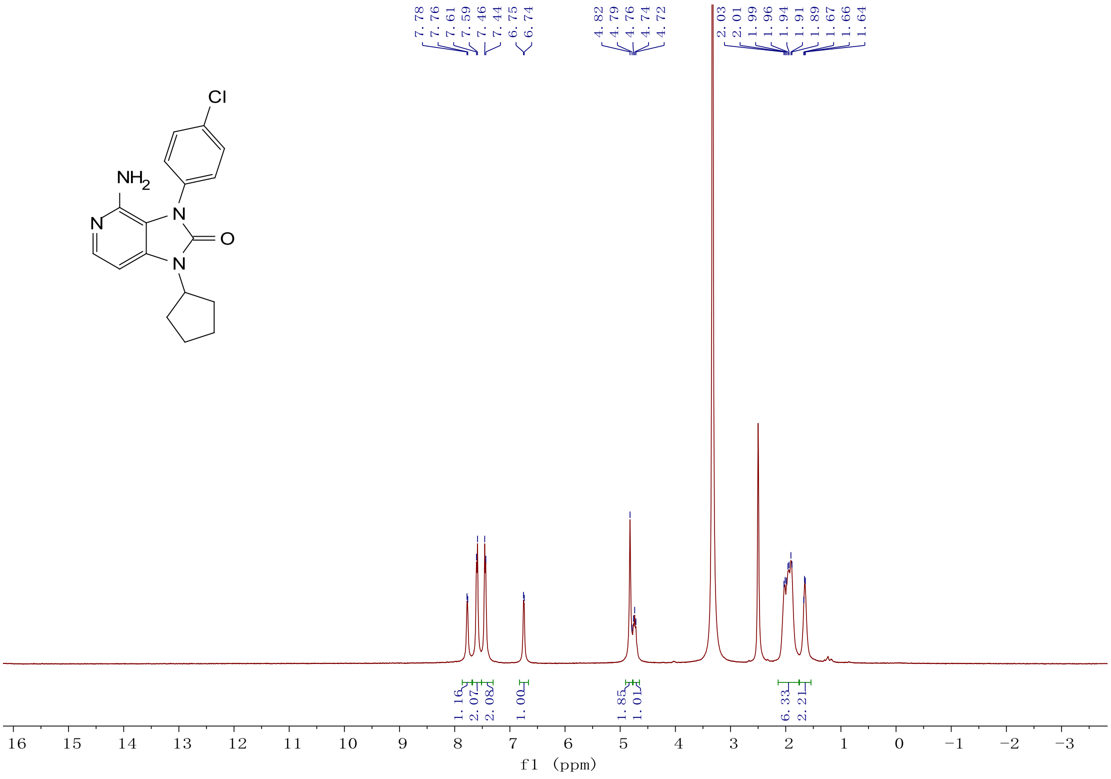

^{13}C NMR Spectrum of Compound **1d**

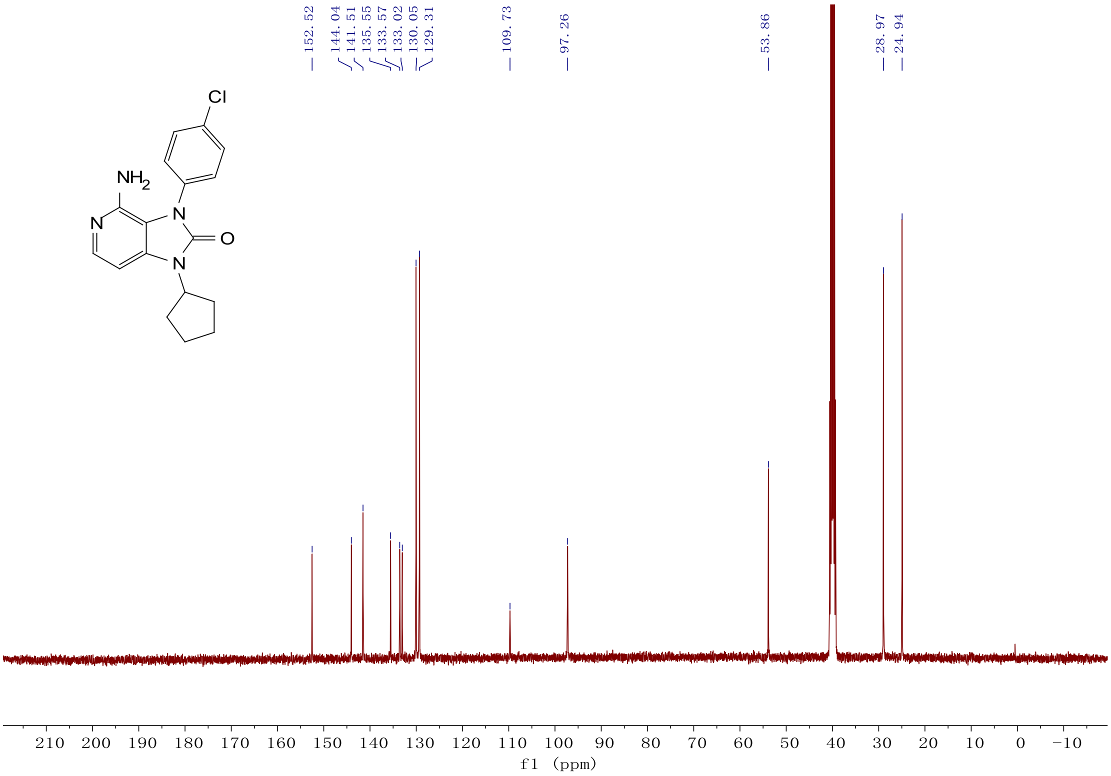

^{1}H NMR Spectrum of Compound **1e**

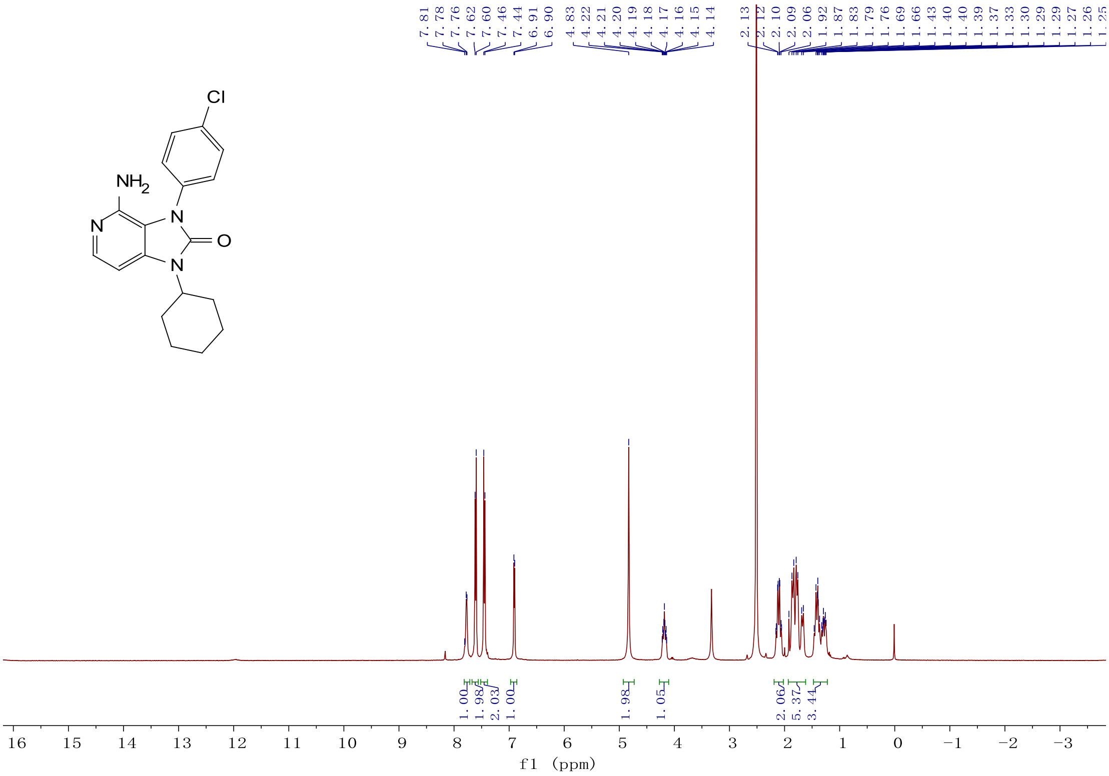

^{13}C NMR Spectrum of Compound **1e**

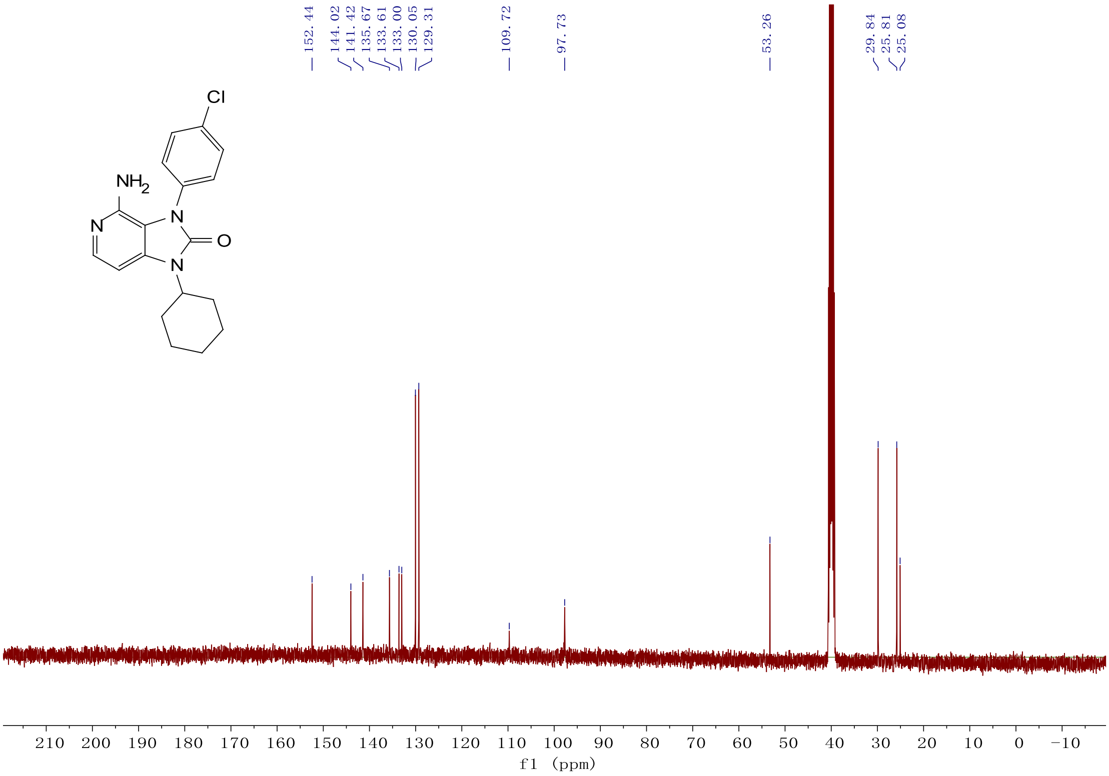

^{1}H NMR Spectrum of Compound **1f**

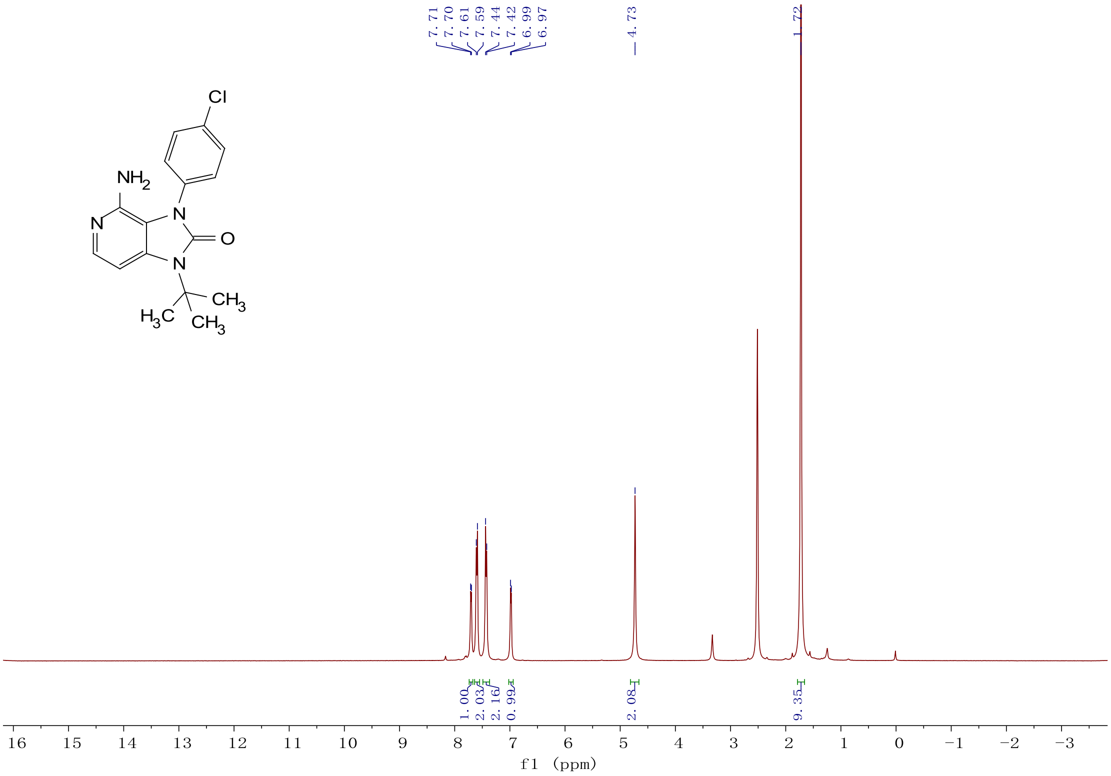

^{13}C NMR Spectrum of Compound **1f**

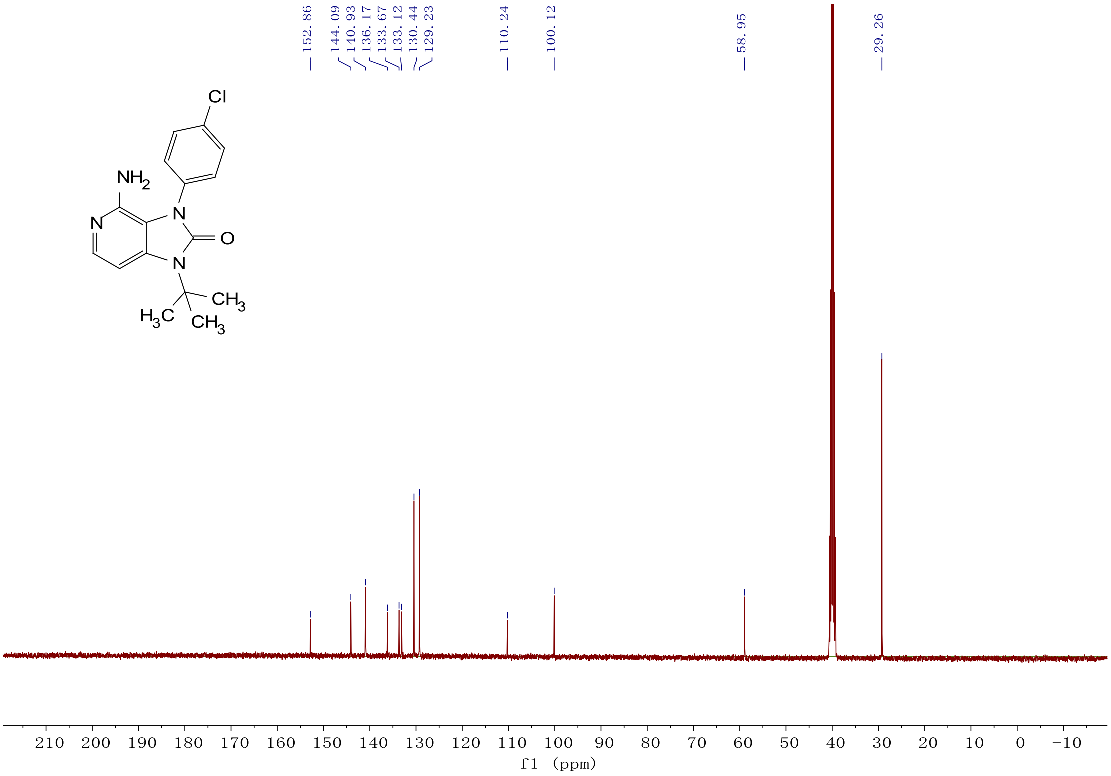

^{1}H NMR Spectrum of Compound **1g**

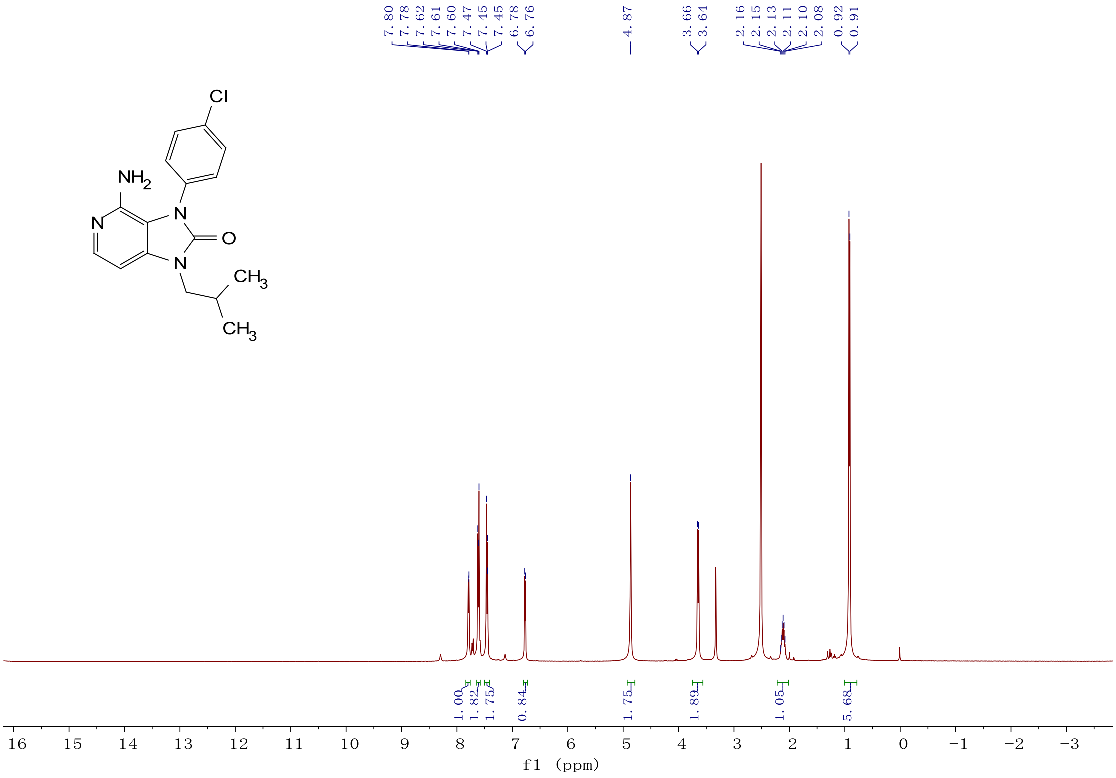

^{13}C NMR Spectrum of Compound **1g**

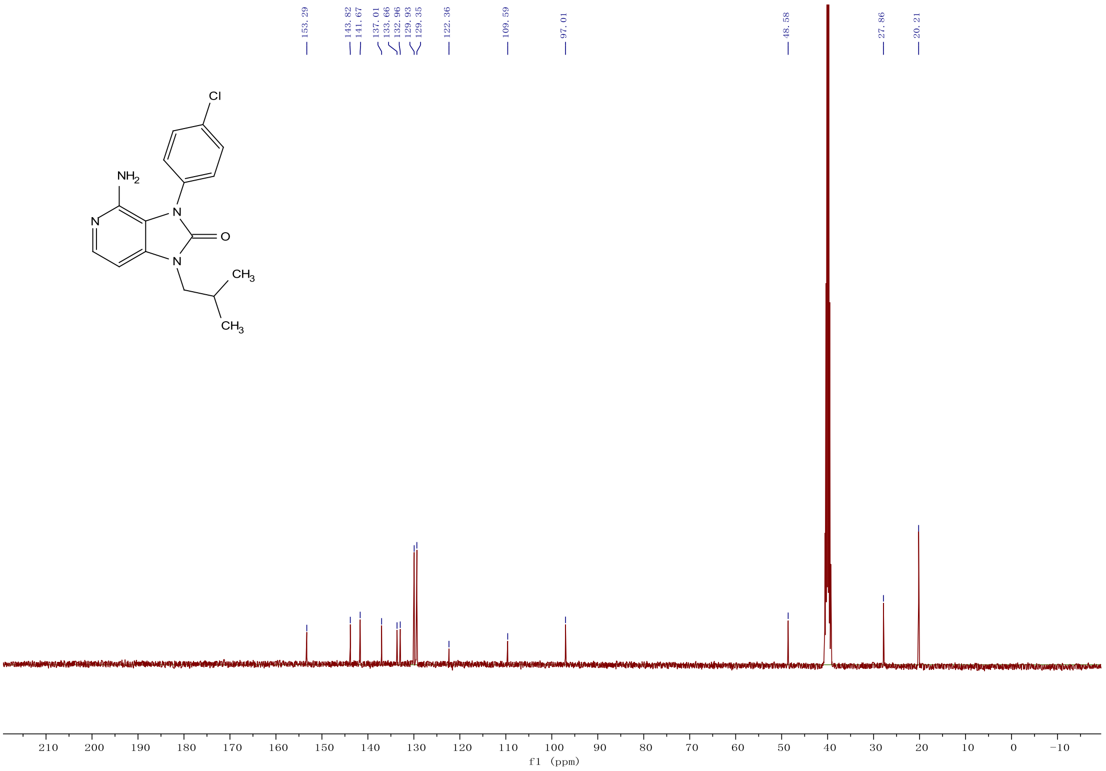

^{1}H NMR Spectrum of Compound **1h**

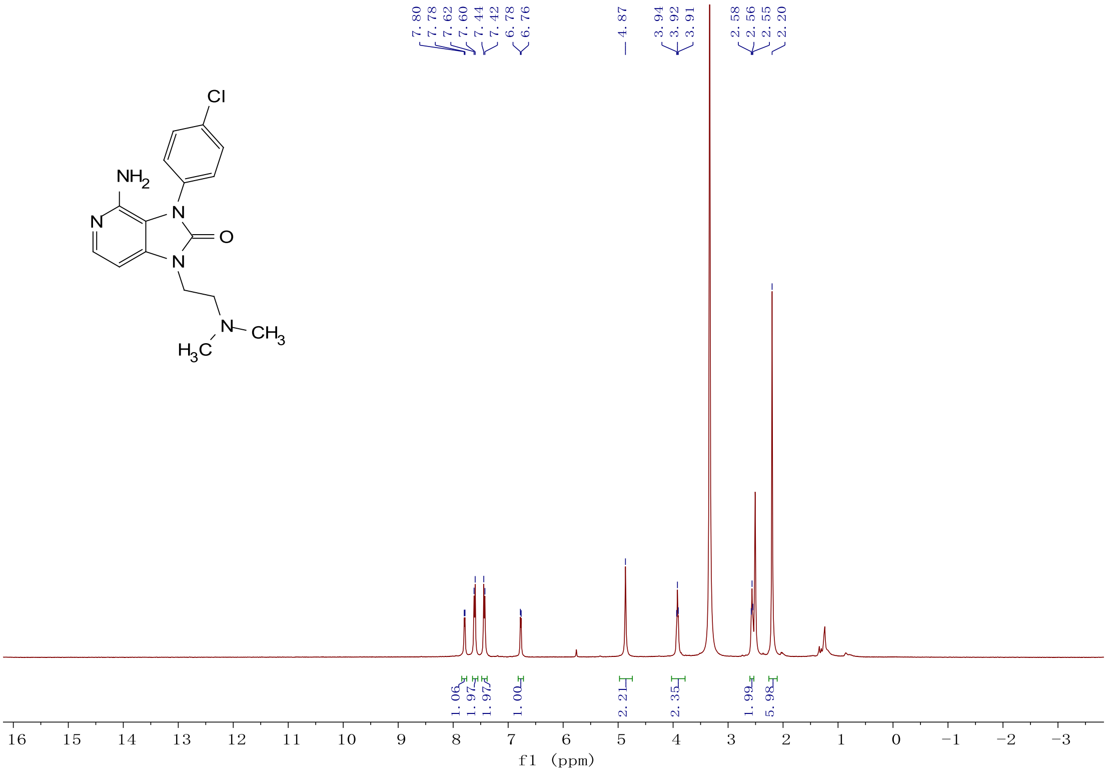

^{13}C NMR Spectrum of Compound **1h**

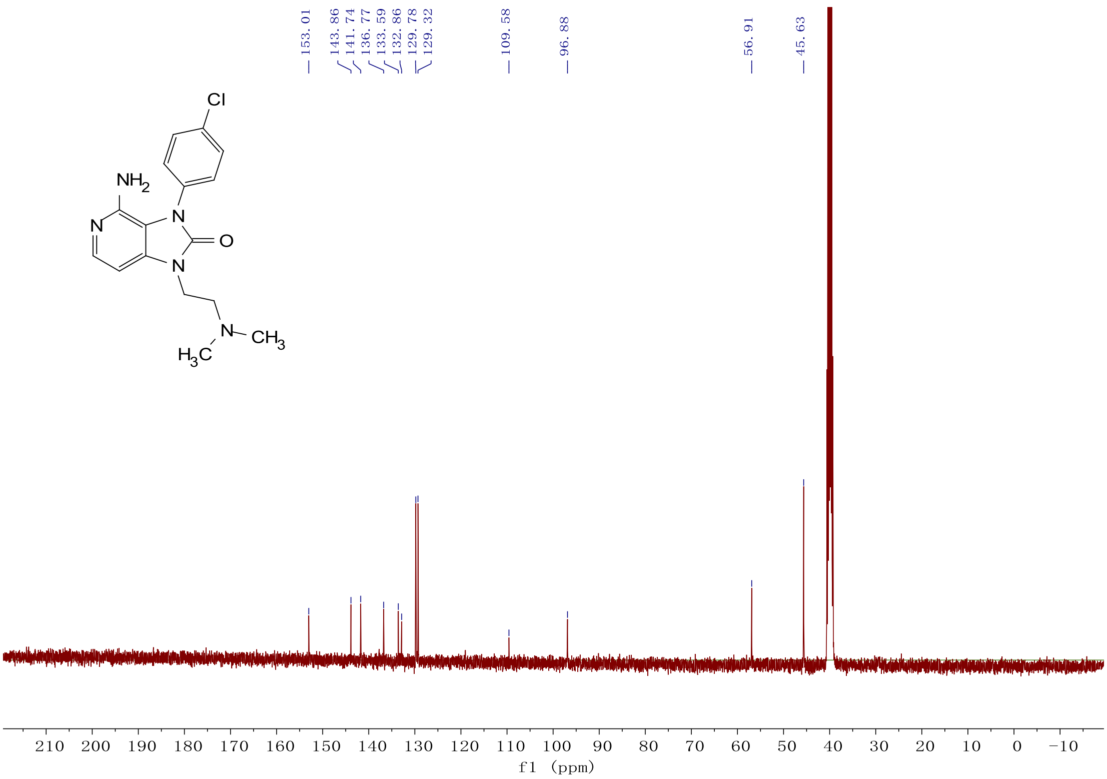

^{1}H NMR Spectrum of Compound **1i**

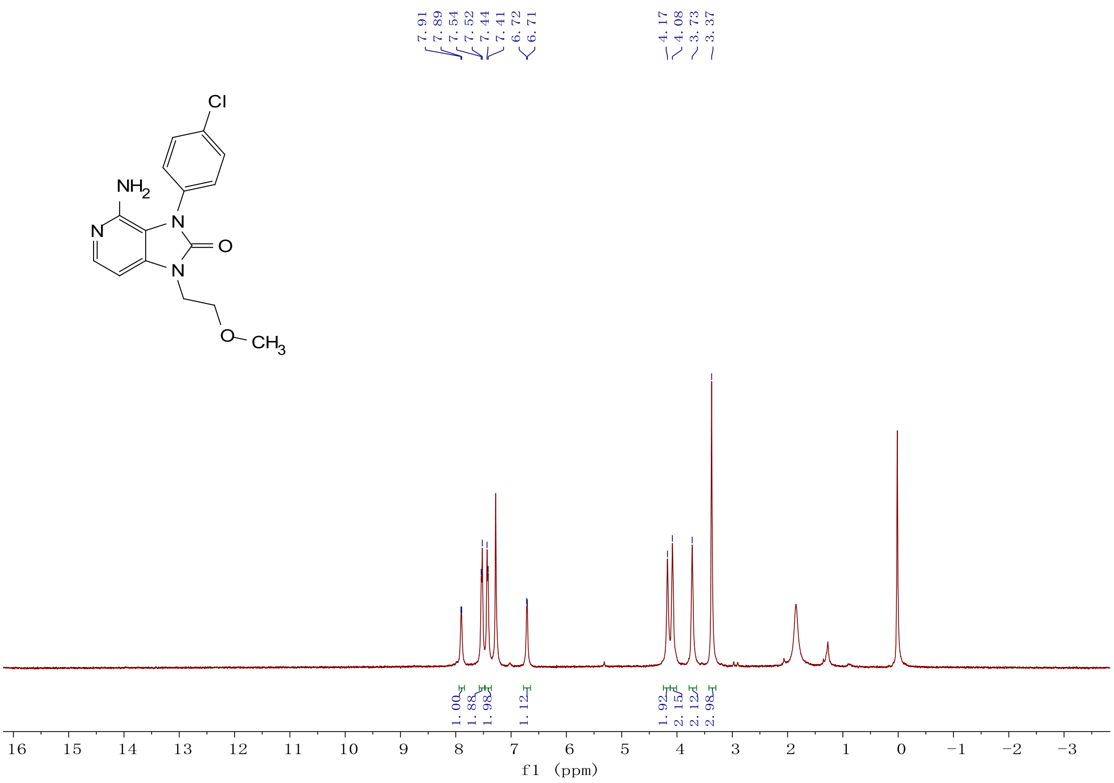

^{13}C NMR Spectrum of Compound **1i**

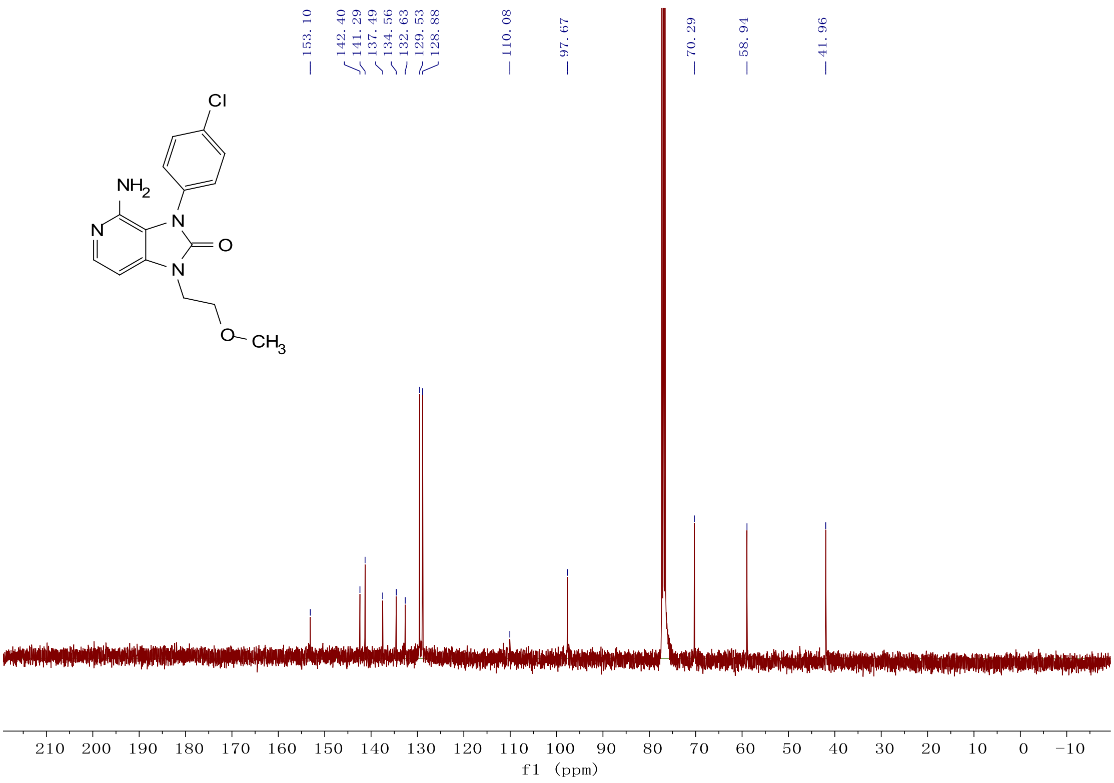

^{1}H NMR Spectrum of Compound **1j**

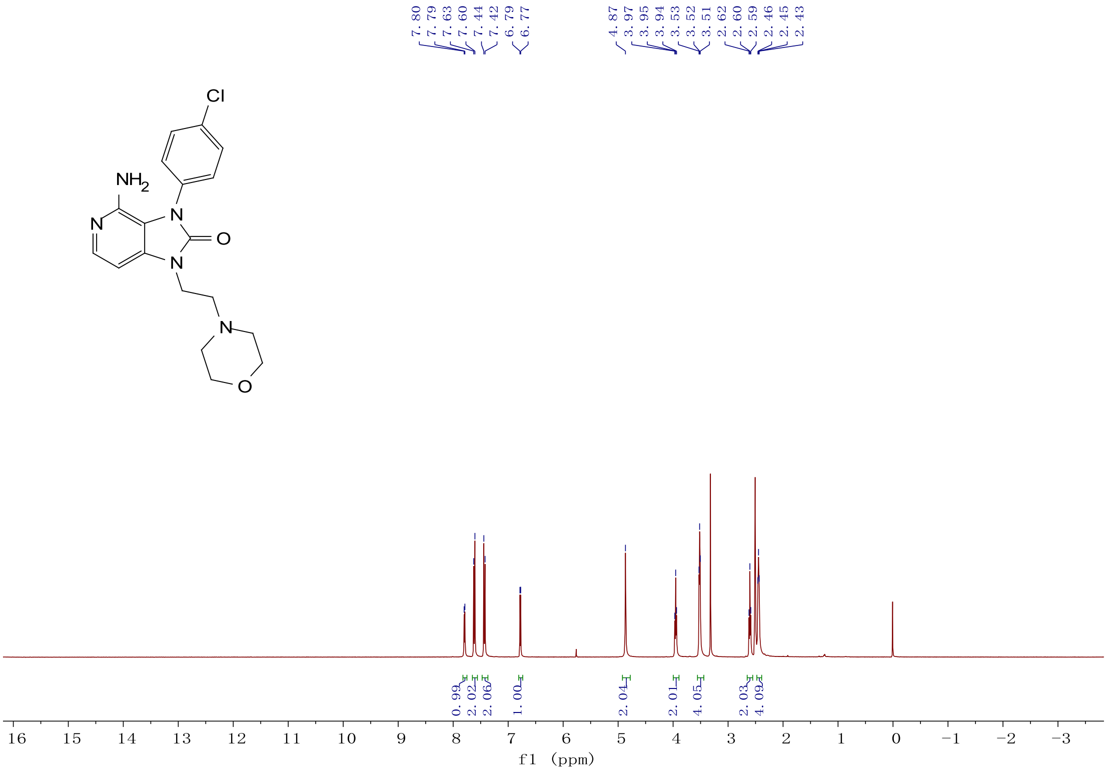

^{13}C NMR Spectrum of Compound **1j**

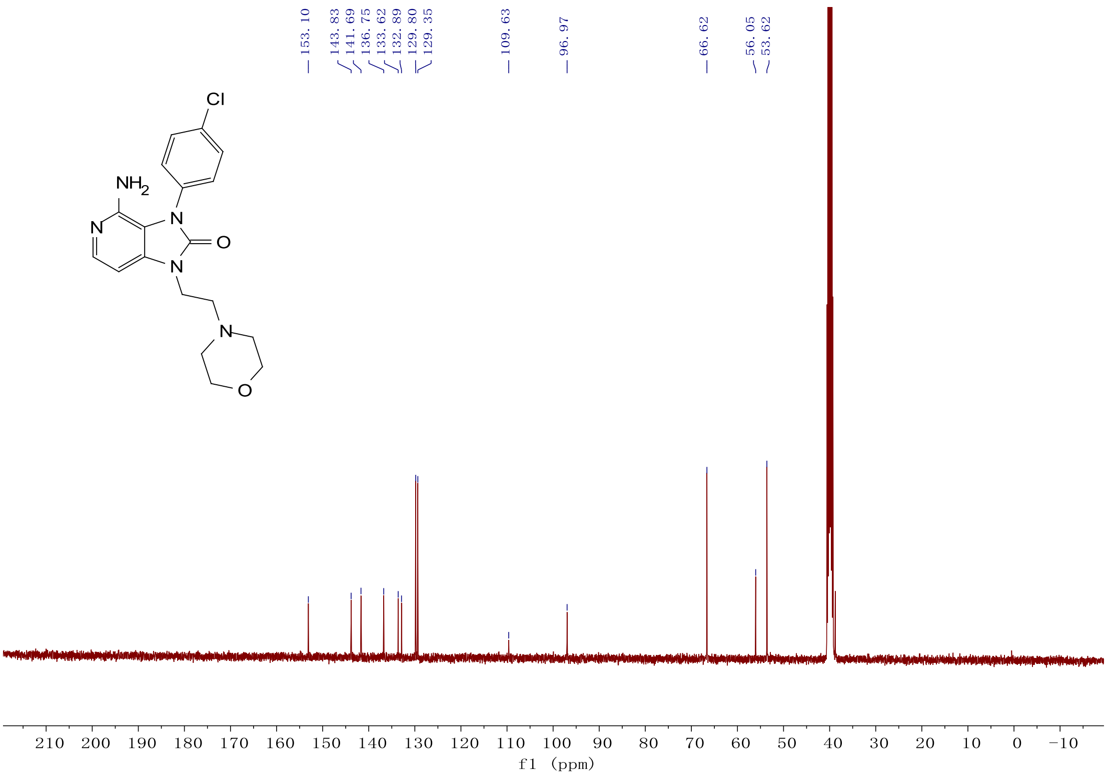

^{1}H NMR Spectrum of Compound **1k**

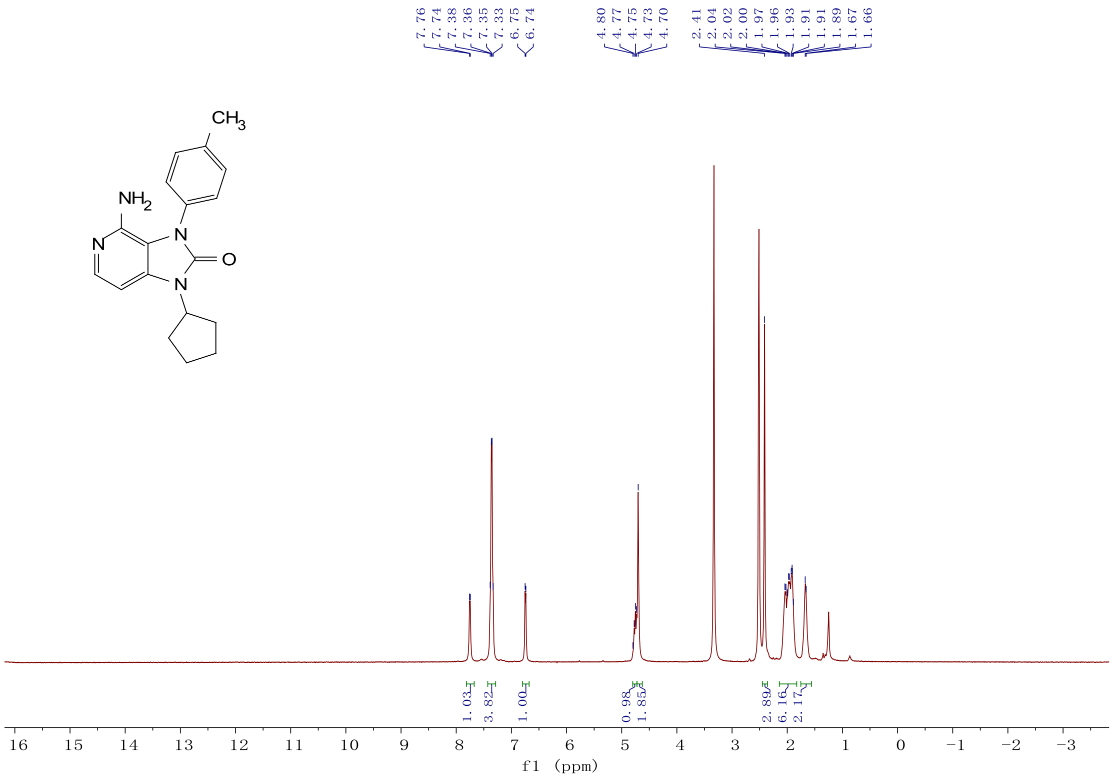

^{13}C NMR Spectrum of Compound **1k**

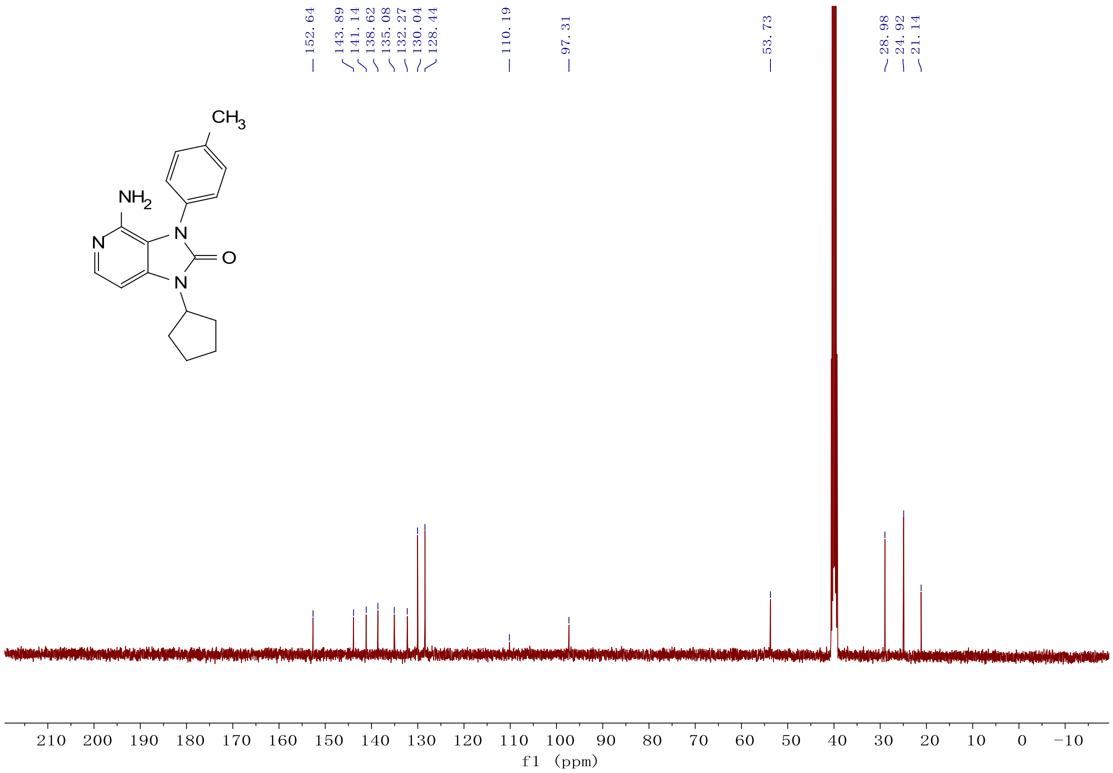

^{1}H NMR Spectrum of Compound **1l**

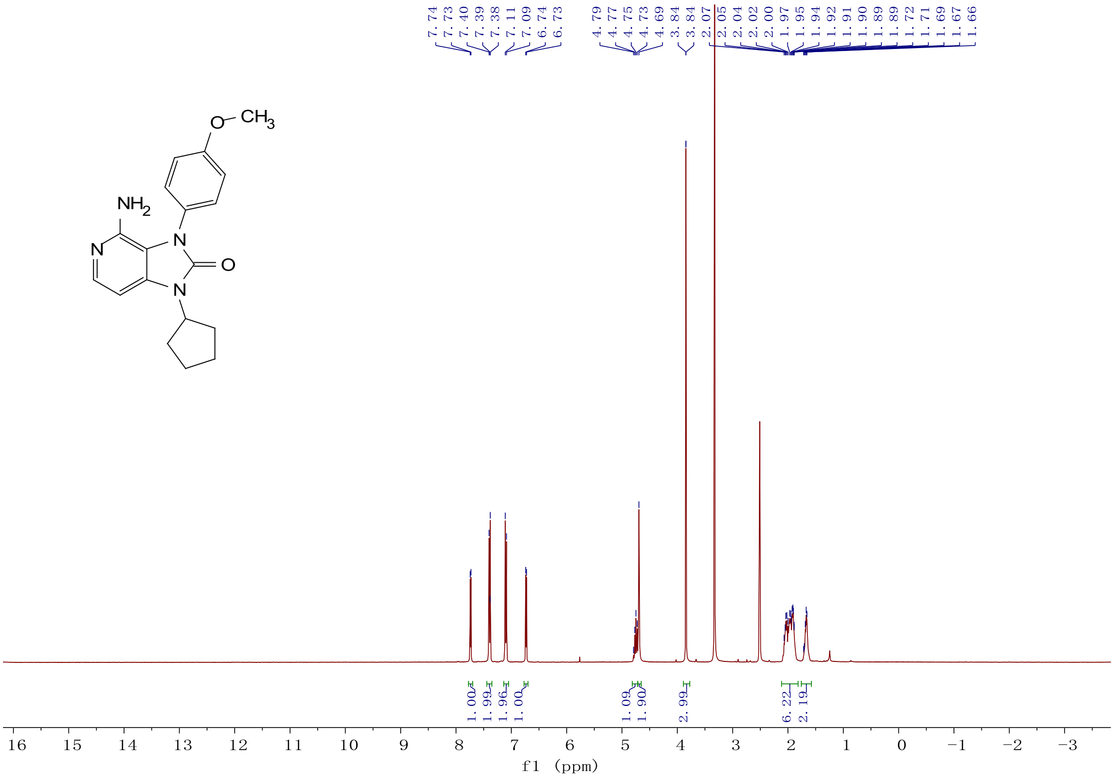

^{13}C NMR Spectrum of Compound **1l**

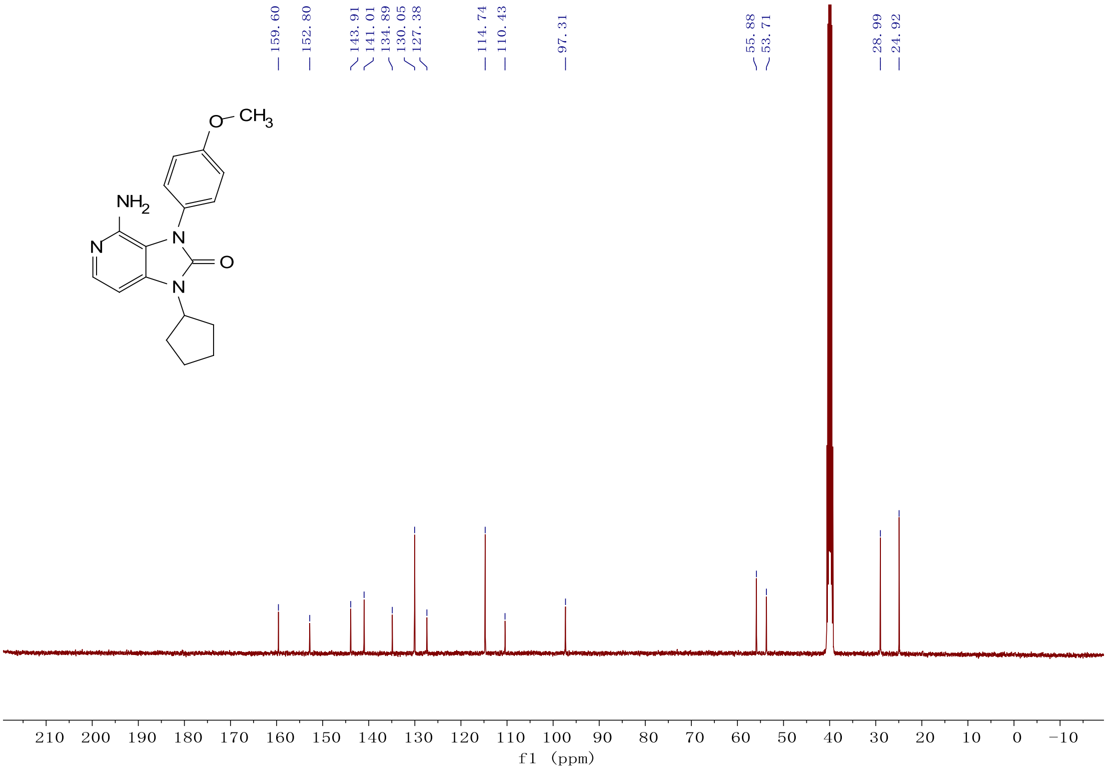

^{1}H NMR Spectrum of Compound **1m**

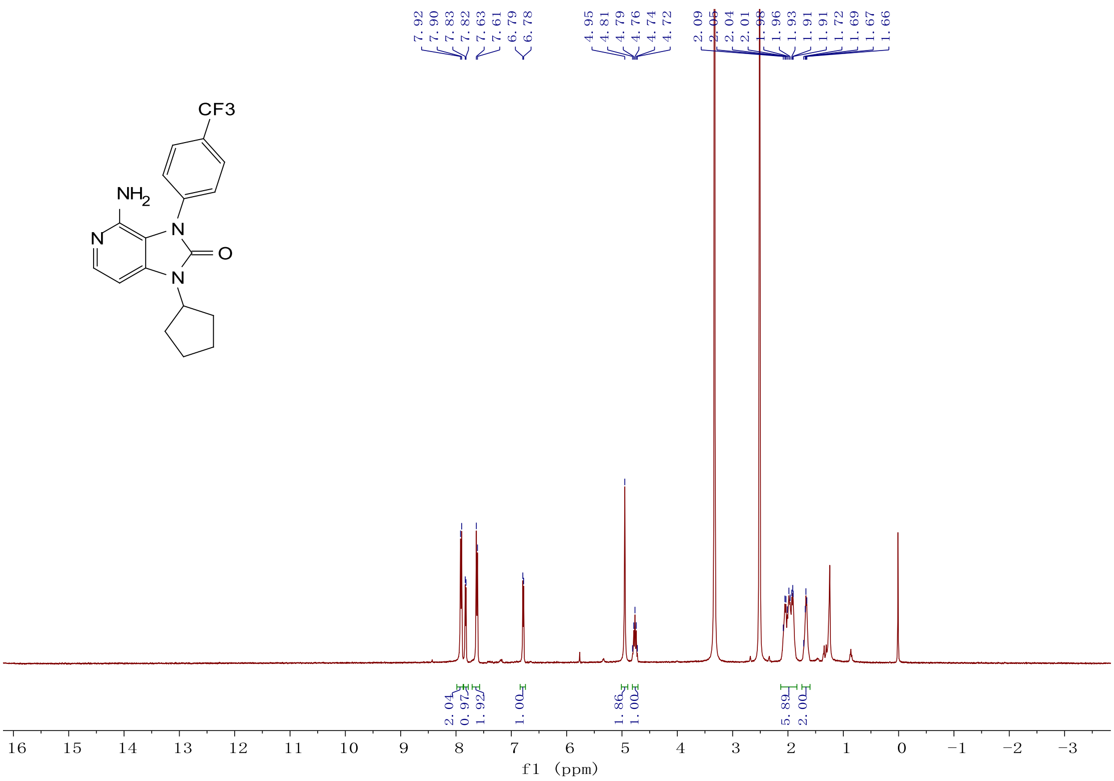

^{13}C NMR Spectrum of Compound **1m**

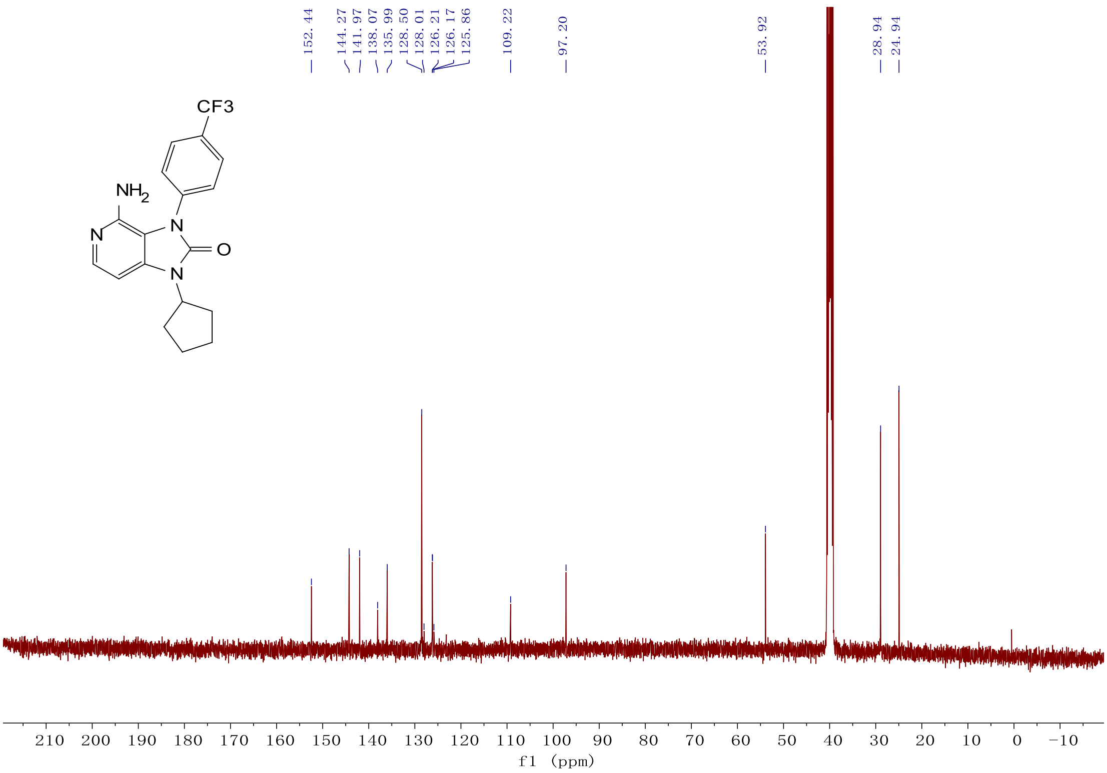

^{1}H NMR Spectrum of Compound **1n**

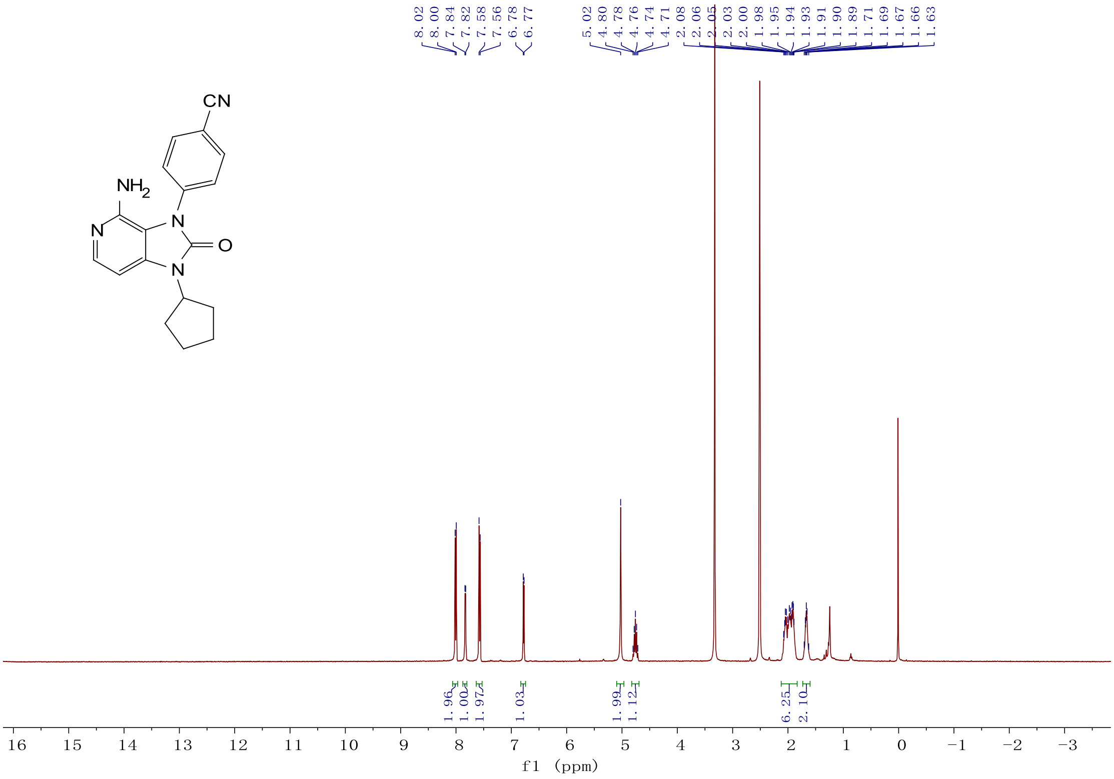

^{13}C NMR Spectrum of Compound **1n**

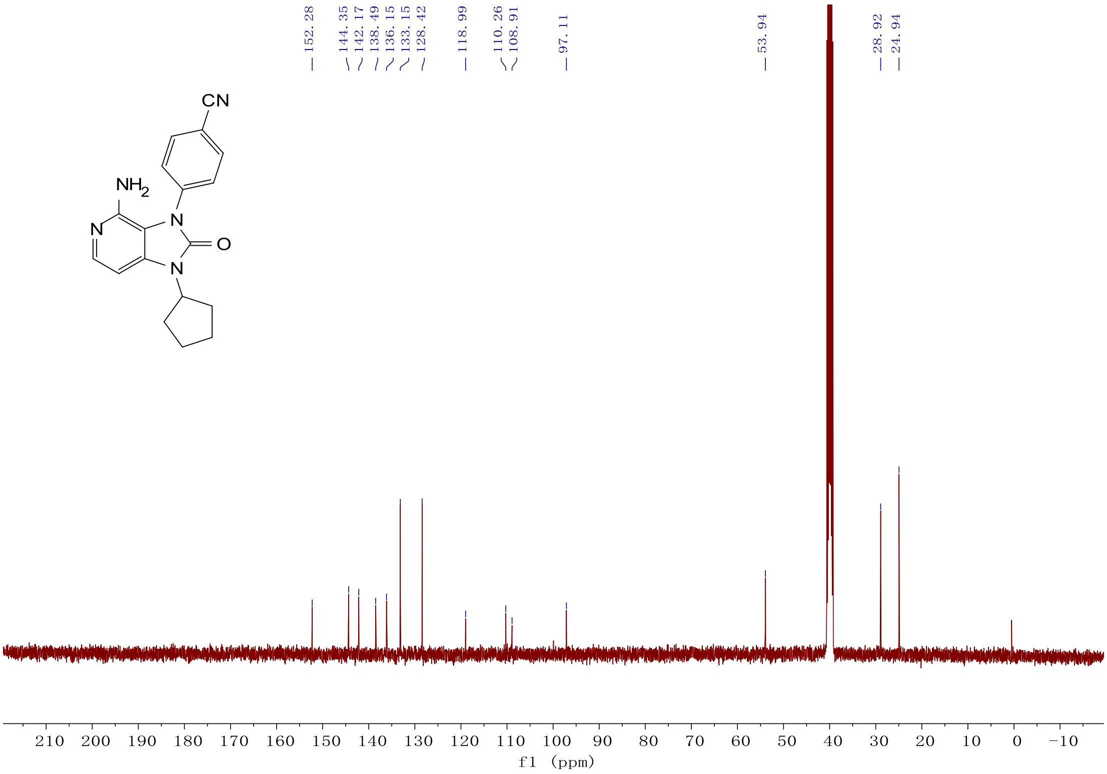

^{1}H NMR Spectrum of Compound **1o**

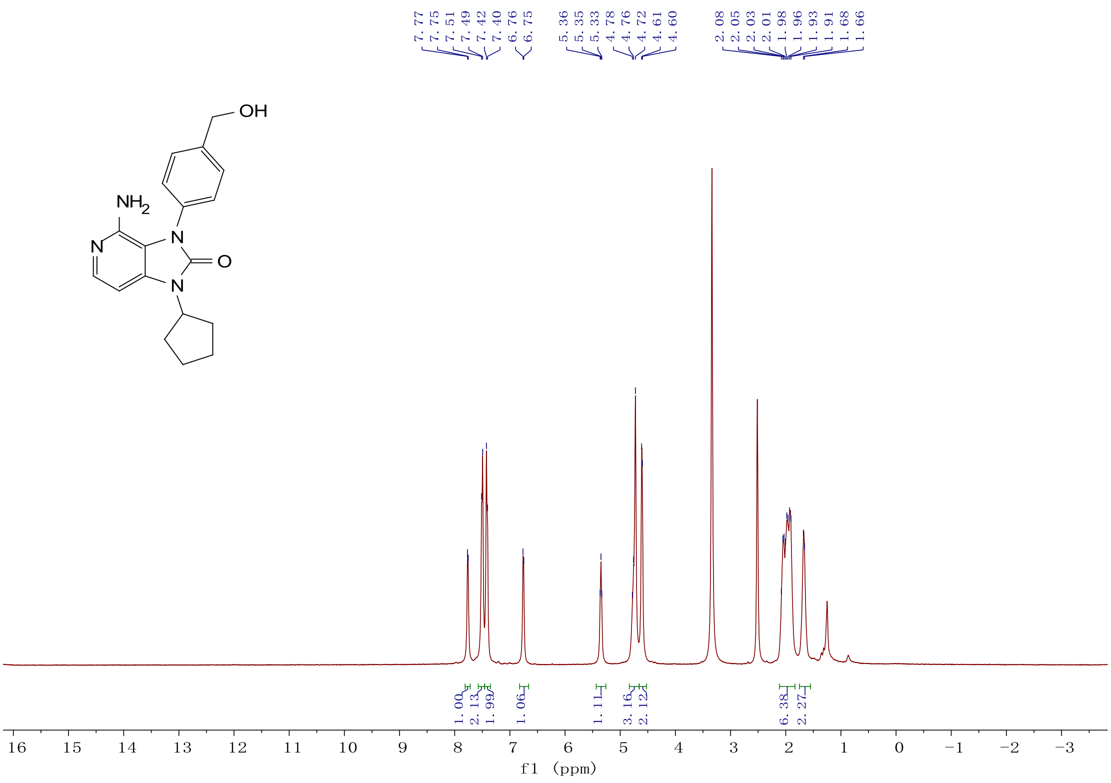

^{13}C NMR Spectrum of Compound **1o**

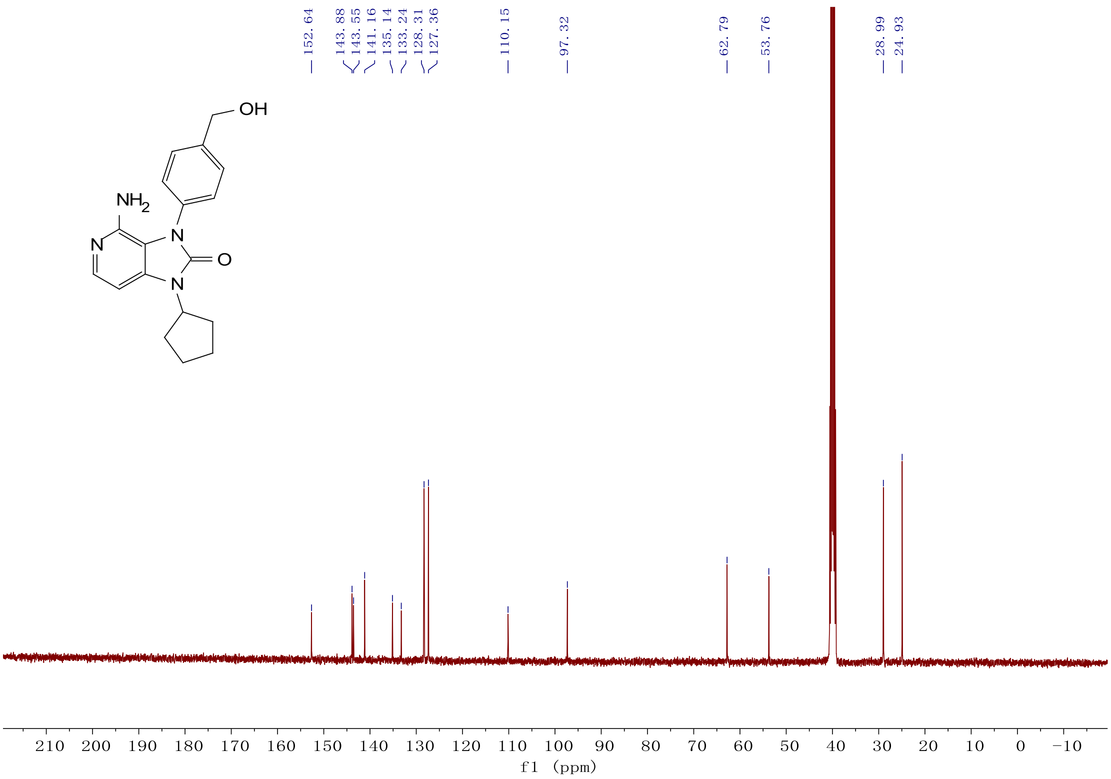

^{1}H NMR Spectrum of Compound **1p**

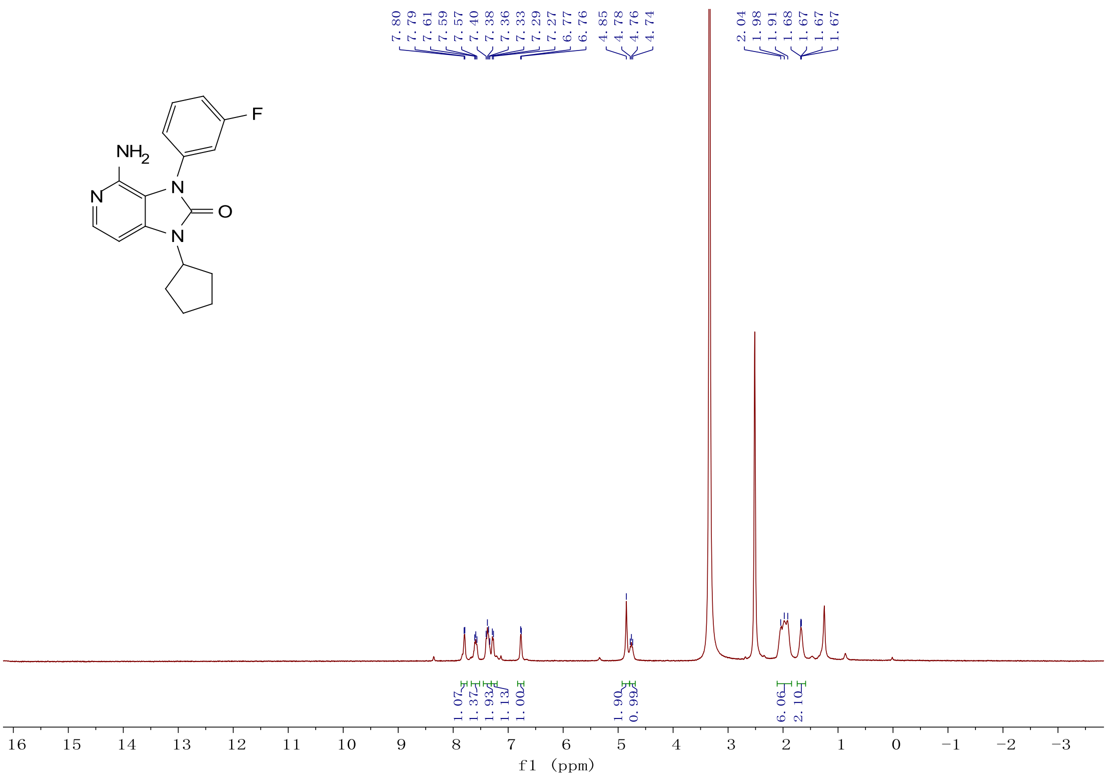

^{13}C NMR Spectrum of Compound **1p**

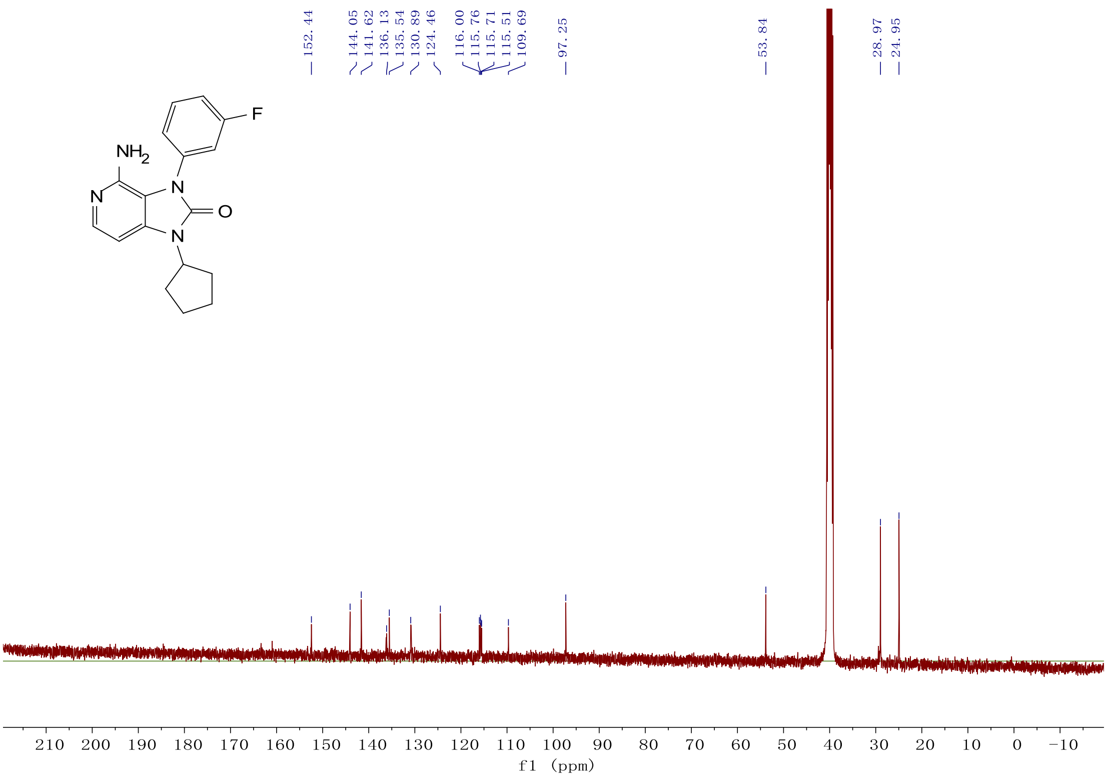

^{1}H NMR Spectrum of Compound **1q**

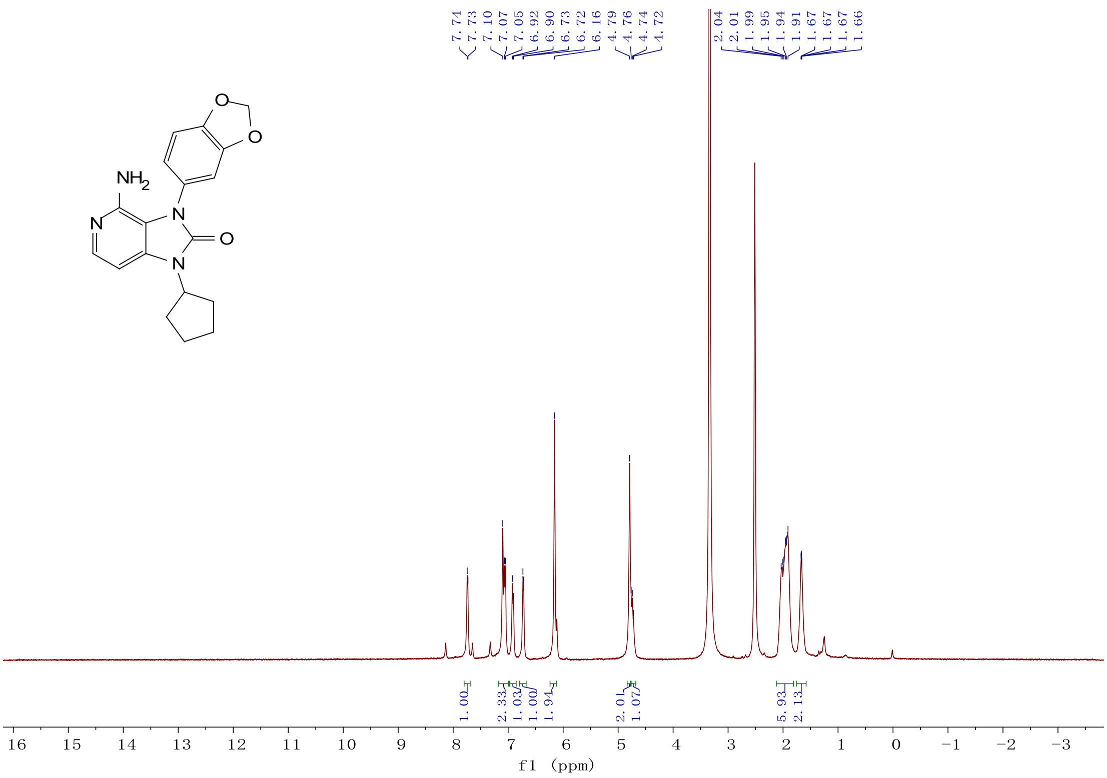

^{13}C NMR Spectrum of Compound **1q**

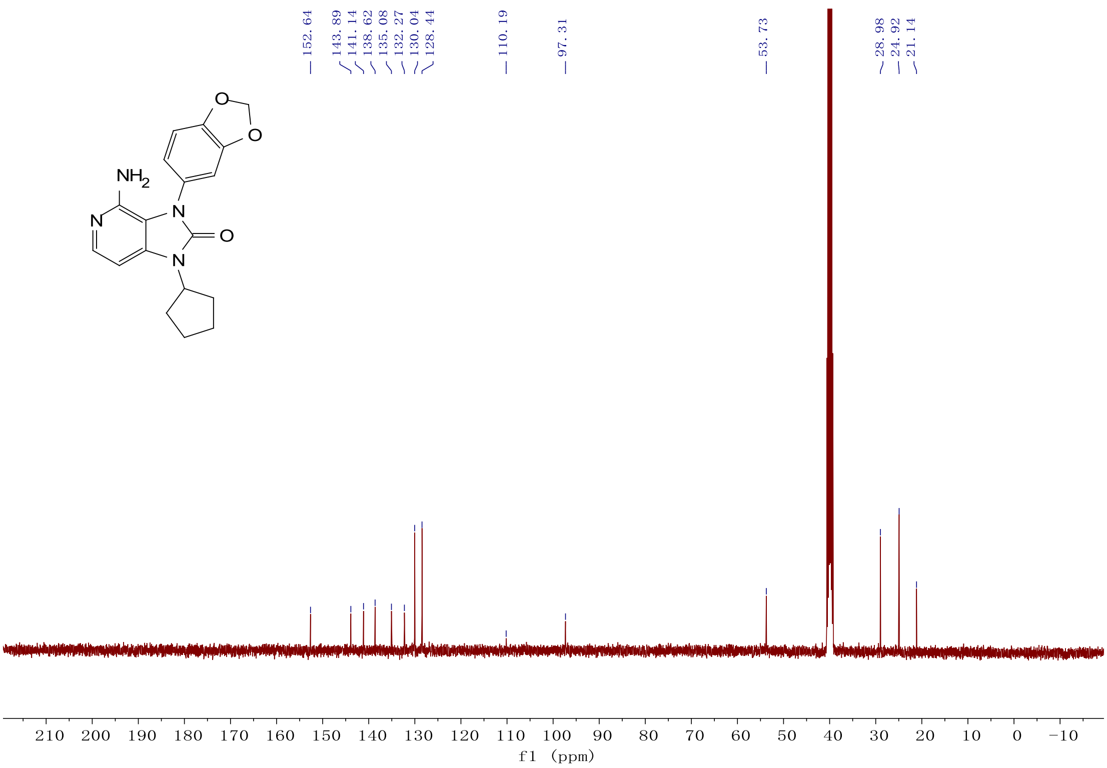

^{1}H NMR Spectrum of Compound **1r**

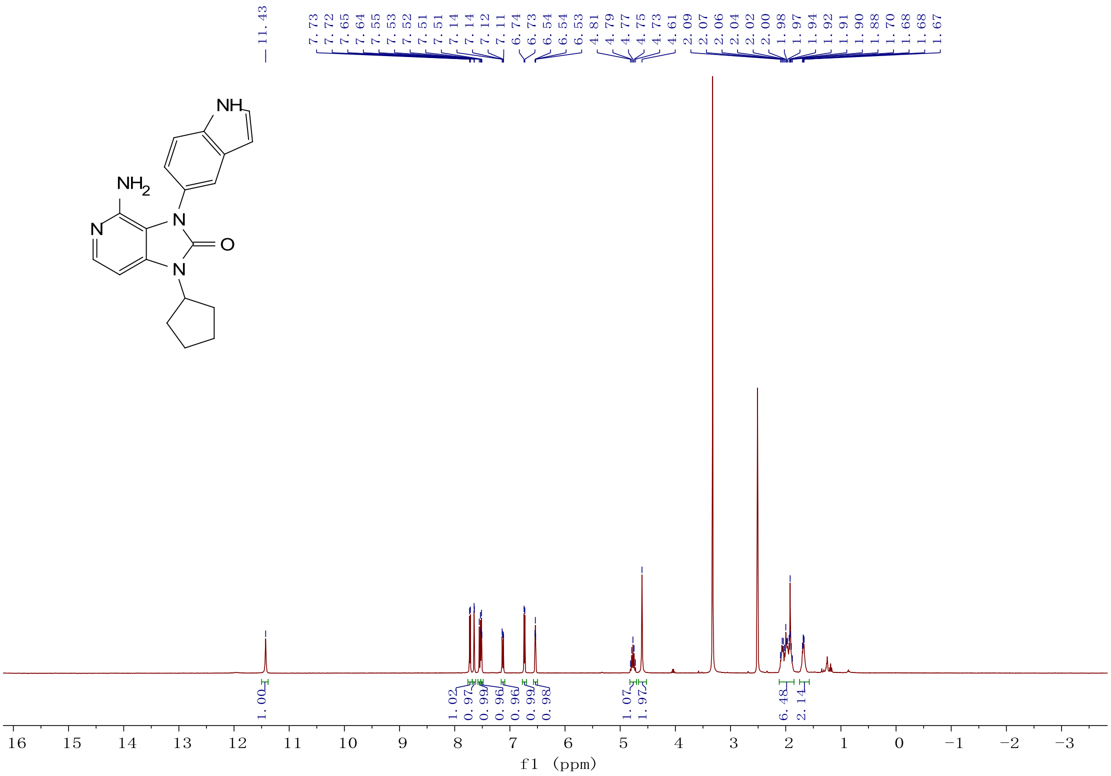

^{13}C NMR Spectrum of Compound **1r**

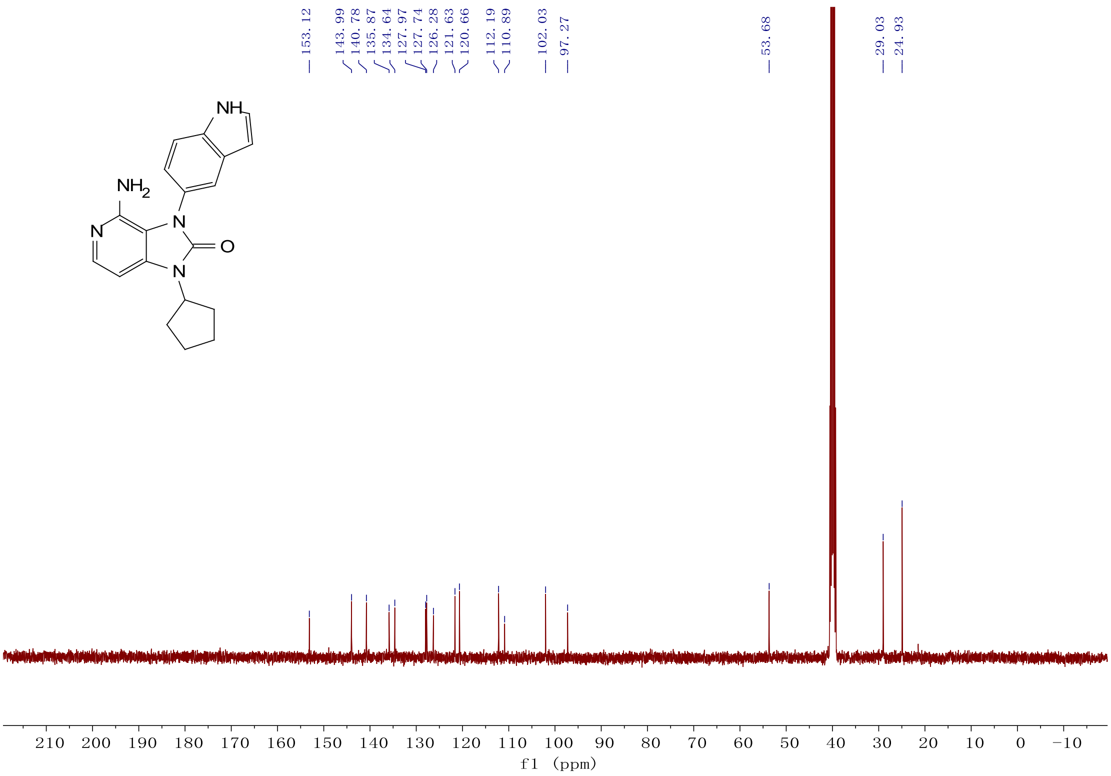

^{1}H NMR Spectrum of Compound **1s**

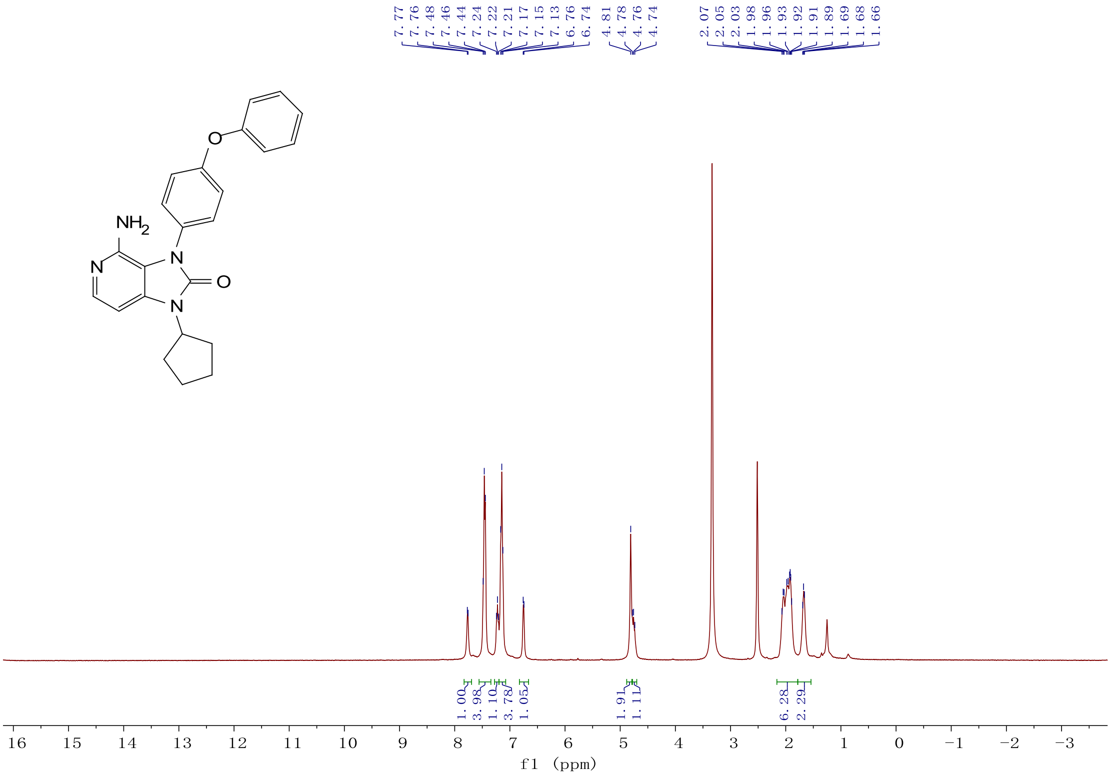

^{13}C NMR Spectrum of Compound **1s**

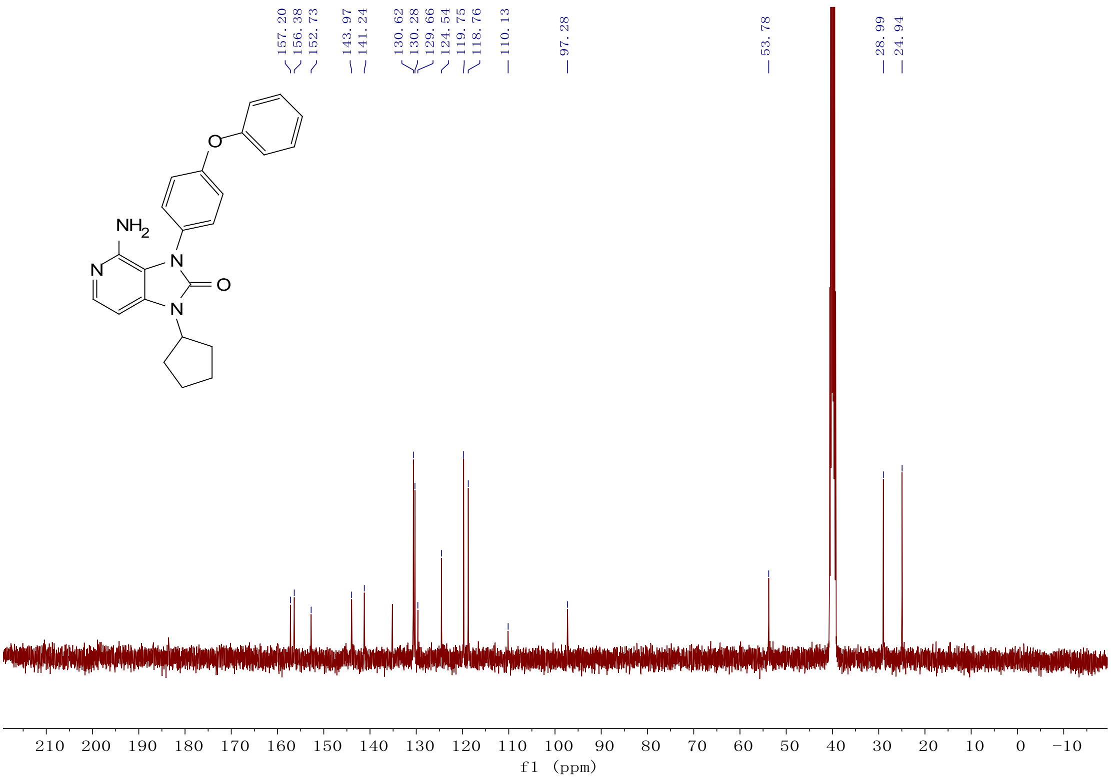

Molecular modeling

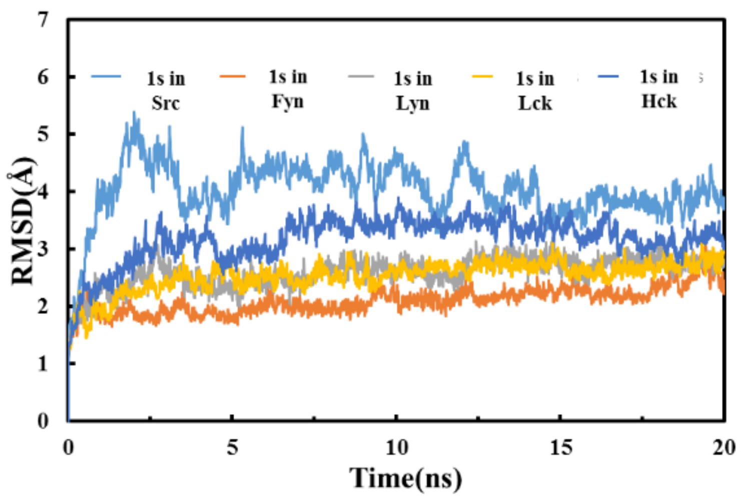

Figure S1. The Root-mean-square deviation (RMSD) monitoring of compounds **1s** in Src, Fyn, Lyn, Lck, Hck.

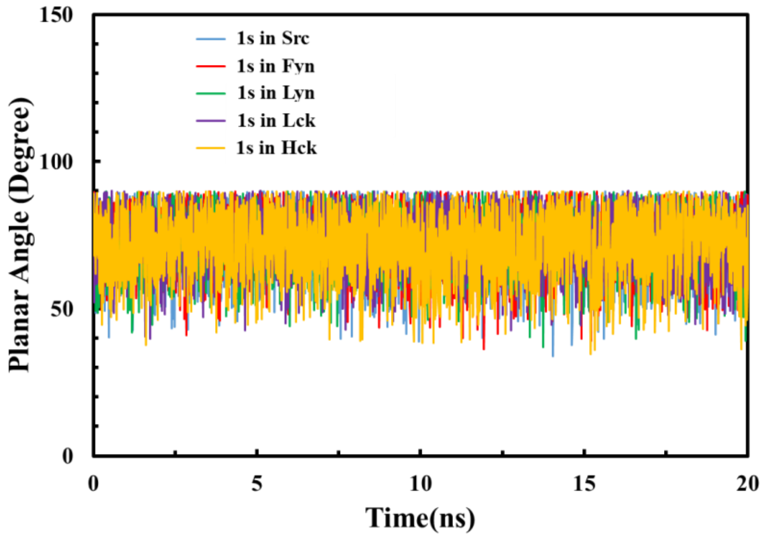

Figure S2. The time-dependent change of planar angle between cyclopentyl group and imidazolone ring in compound **1s** interacted with Src, Fyn, Lyn, Lck, and Hck.

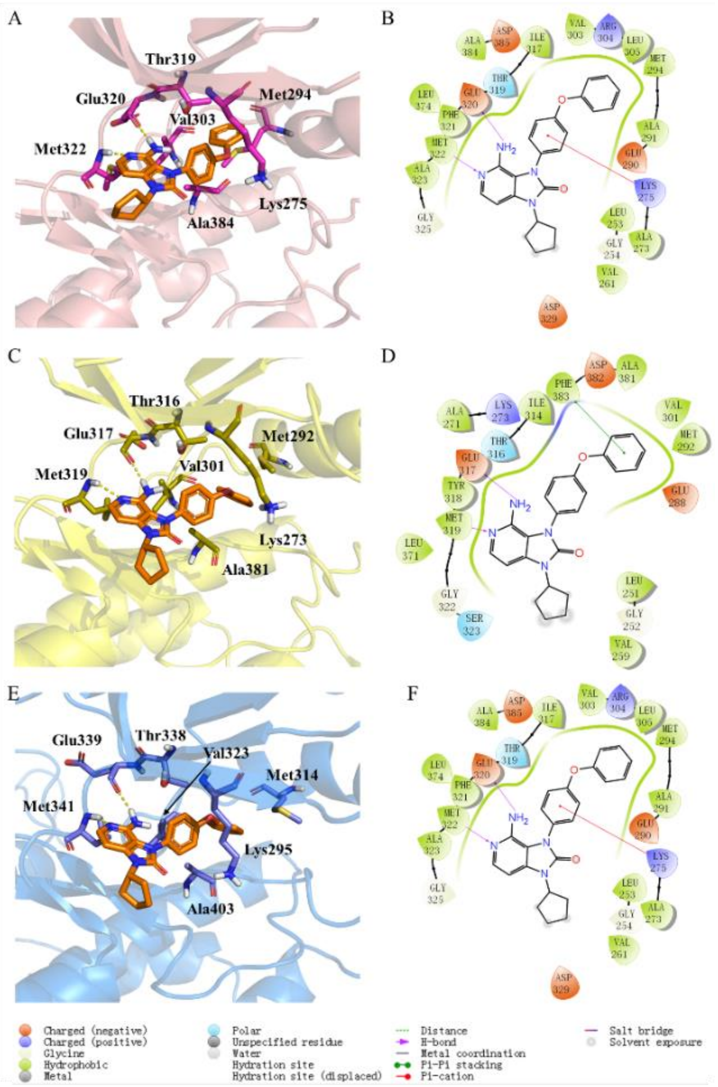

Figure S3. Representative structure of compound **1s** interacted with Lyn (A), Lck (C), and Hck (E). The proteins were displayed as ribbon, and the interacted residues were exhibited as stick (magenta for Lyn, yellow for Lck, and Blue for Hck). Compound **1s** was showed as orange stick, and the hydrogen bonds were displayed as yellow dashes. 2D diagram of **1s** interacted with Lyn (B), Lck (D), and Hck (F).

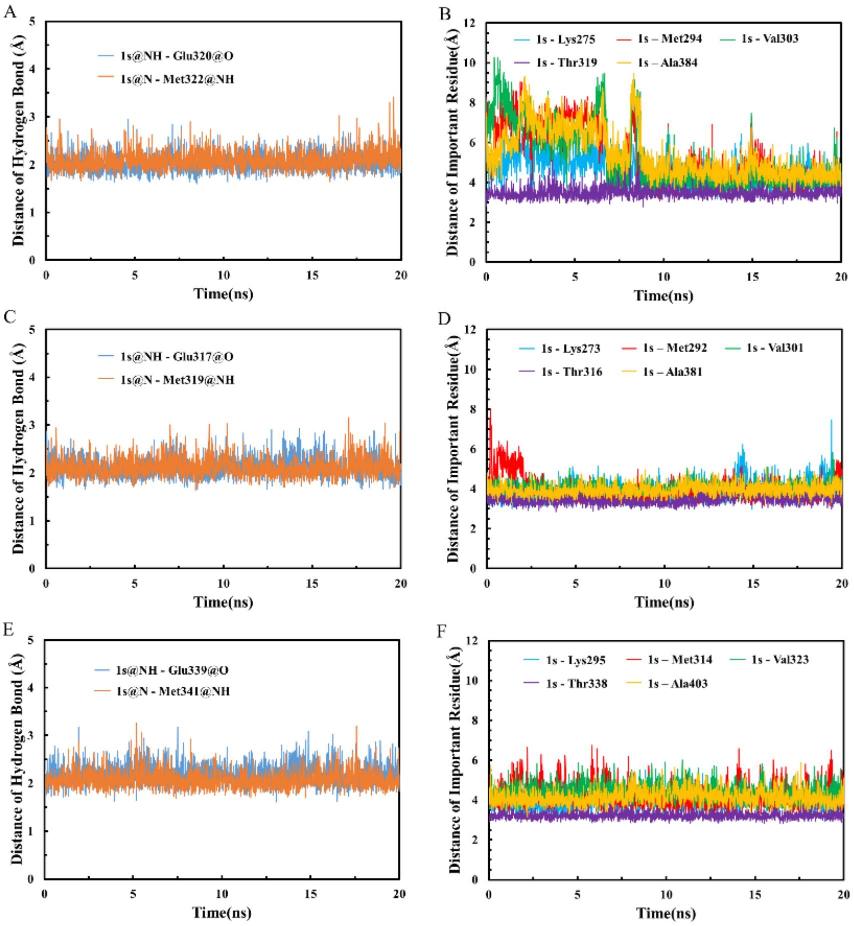

Figure S4. The time-dependent change of hydrogen bond length between compound **1s** and residues in Lyn (A), Lck (C) and Hck (E). The time-dependent change of distance between compound **1s** and important residues in Lyn (B), Lck (D) and Hck (F).

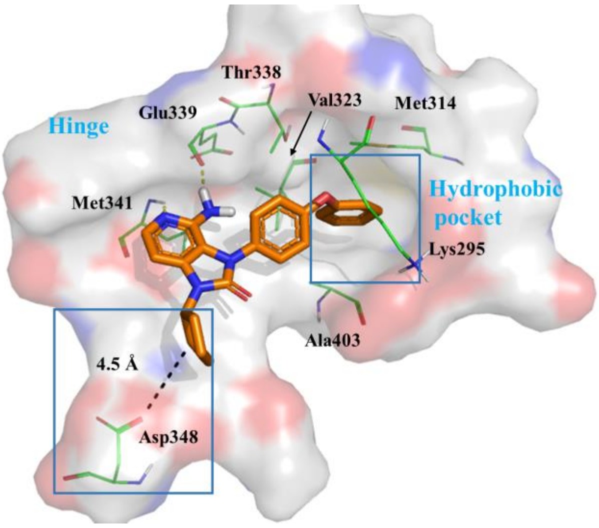

Figure S5. Compound **1s** binding with the ATP pocket in Src based on MD simulation. The ligand was displayed as orange stick and the interacted residues were showed as

green line. The yellow dash represents hydrogen bond and the black dash represents the distance between Asp348 and the ligand. The areas targeted for modification strategies are indicated by rectangles.

Table S1. ADME parameters simulation of target compounds

**Principal Descriptors**	**PP2**	**1d**	**1e**	**1q**	**1s**	**Dasatinib**	**Standard Range** [a]
QPlogPo/w [b]	3.54	3.83	4.11	2.93	5.60	2.88	-2.0 - 6.5
QPlogKhsa [c]	0.37	0.54	0.67	0.29	0.90	0.16	-1.5 - 1.5
QPlogBB [d]	-0.09	-0.12	-0.17	-0.24	-0.64	-0.96	-3.0 - 1.2
#metab [e]	1	2	2	2	2	6	1 - 8
CNS	0	0	0	0	0	-1	-2 (inactivity), +2 (activity)
QPloghERG [f]	-4.92	-5.14	-5.22	-4.63	-6.17	-7.38	< -5
QPPCaco [g]	1824	1558	1399	1562	1019	105	< 25 poor, > 500 great
QPPMDCK [h]	2339	1972	1756	801	505	135	< 25 poor, > 500 great

[a] Statistics of 95% of known drugs according to Qikprop (Schrödinger@ 2017).

[b] QPlogPo/w, octanol-water partitioning coefficient.

[c] QplogKhsa, binding to human serum albumin.

[d] QplogBB, brain/blood partition coefficient.

[e] #metab, number of likely metabolic reactions.

[f] QPloghERG, predicted IC_{50} value for blockage of hERG K^+ channels.

[g] QPPCaco, predicted apparent Caco-2 cell permeability in nm/sec.

[h] QPPMDCK, predicted apparent MDCK cell permeability in nm/sec.
